# Deciphering the Transcriptional Metabolic Profile of Adipose-Derived Stem Cells During Osteogenic Differentiation and Epigenetic Drug Treatment

**DOI:** 10.3390/cells14020135

**Published:** 2025-01-17

**Authors:** Giulia Gerini, Alice Traversa, Fabrizio Cece, Matteo Cassandri, Paola Pontecorvi, Simona Camero, Giulia Nannini, Enrico Romano, Francesco Marampon, Mary Anna Venneri, Simona Ceccarelli, Antonio Angeloni, Amedeo Amedei, Cinzia Marchese, Francesca Megiorni

**Affiliations:** 1 Department of Experimental Medicine, Sapienza University of Rome, 00161 Rome, Italy; giulia.gerini@uniroma1.it (G.G.); fabrizio.cece@uniroma1.it (F.C.); matteo.cassandri@uniroma1.it (M.C.); paola.pontecorvi@uniroma1.it (P.P.); maryanna.venneri@uniroma1.it (M.A.V.); simona.ceccarelli@uniroma1.it (S.C.); antonio.angeloni@uniroma1.it (A.A.); cinzia.marchese@uniroma1.it (C.M.); 2 Department of Life Sciences, Health and Health Professions, Link Campus University, 00165 Rome, Italy; a.traversa@unilink.it (A.T.); s.camero@unilink.it (S.C.); 3 Department of Experimental and Clinical Medicine, University of Florence, 50121 Florence, Italy; giulia.nannini@unifi.it (G.N.); amedeo.amedei@unifi.it (A.A.); 4 Department of Sense Organs, Sapienza University of Rome, 00161 Rome, Italy; enrico.romano@uniroma1.it; 5 Department of Radiological, Oncological and Pathological Sciences, Sapienza University of Rome, 00161 Rome, Italy; francesco.marampon@uniroma1.it

**Keywords:** adipose-derived stem cells, osteogenic differentiation, DNA methyltransferase inhibitor, metabolic pathways

## Abstract

Adipose-derived mesenchymal stem cells (ASCs) are commonly employed in clinical treatment for various diseases due to their ability to differentiate into multi-lineage and anti-inflammatory/immunomodulatory properties. Preclinical studies support their use for bone regeneration, healing, and the improvement of functional outcomes. However, a deeper understanding of the molecular mechanisms underlying ASC biology is crucial to identifying key regulatory pathways that influence differentiation and enhance regenerative potential. In this study, we employed the NanoString nCounter technology, an advanced multiplexed digital counting method of RNA molecules, to comprehensively characterize differentially expressed transcripts involved in metabolic pathways at distinct time points in osteogenically differentiating ASCs treated with or without the pan-DNMT inhibitor RG108. *In silico* annotation and gene ontology analysis highlighted the activation of ethanol oxidation, ROS regulation, retinoic acid metabolism, and steroid hormone metabolism, as well as in the metabolism of lipids, amino acids, and nucleotides, and pinpointed potential new osteogenic drivers like AOX1 and ADH1A. RG108-treated cells, in addition to the upregulation of the osteogenesis-related markers RUNX2 and ALPL, showed statistically significant alterations in genes implicated in transcriptional control (MYCN, MYB, TP63, and IRF1), ethanol oxidation (ADH1C, ADH4, ADH6, and ADH7), and glucose metabolism (SLC2A3). These findings highlight the complex interplay of the metabolic, structural, and signaling pathways that orchestrate osteogenic differentiation. Furthermore, this study underscores the potential of epigenetic drugs like RG108 to enhance ASC properties, paving the way for more effective and personalized cell-based therapies for bone regeneration.

## 1. Introduction

Bone defects represent a significant challenge in modern medicine, affecting millions of patients worldwide and presenting complex issues in terms of treatment and recovery. These defects can arise from various causes, including traumatic injuries, congenital abnormalities, infections, inflammatory and degenerative diseases (such as osteoarthritis), reduced bone density (e.g., osteoporosis), and tumors (e.g., osteosarcomas), leading to functional impairment and disability [1]. Indeed, bone defects have a profound clinical impact with significant social and economic burdens, as the repair and regeneration of skeletal defects can result in severe functional loss, chronic pain, and thus decreased quality of life for affected individuals. Moreover, these conditions can lead to secondary complications, such as joint stiffness, muscle atrophy, and neurological deficits, further exacerbating the patient’s condition [2]. Traditional treatment approaches often involve surgical interventions, bone grafts, and the use of orthopedic devices. However, these methods have limitations, including the amount of bone that can be harvested, donor site morbidity, and potential immune response, meaning they may not always provide optimal clinical outcomes [3]. Many therapeutic interventions rely on autologous or allogeneic bone grafts. However, autologous bone grafting is associated with several limitations, including donor site morbidity, limited graft availability, and the risk of infection and complications at the graft site. Allogeneic bone grafts, derived from cadaveric or donor sources, offer an alternative to autologous grafts, but carry a risk of immune rejection and disease transmission. Moreover, synthetic bone substitutes and biomaterials, such as calcium phosphate ceramics and bioresorbable polymers, have been developed as alternatives to traditional bone grafts, but their efficacy and long-term outcomes remain under investigation [4,5]. Despite advances in treatment modalities, the management of bone defects remains challenging, especially in cases of large or complex defects where traditional approaches may be ineffective or inadequate. Consequently, there is a growing interest in exploring innovative strategies for enhancing bone repair and regeneration.

In recent years, regenerative medicine has emerged as a promising approach for addressing bone defects, offering the potential to facilitate tissue growth and restore damaged tissues through the use of stem cells, biomaterials, and growth factors that can support bone healing. Among the different types of adult stem cells investigated for bone regeneration, a potential alternative solution to treat osteochondral diseases and fractures involves the use of stromal/stem cells. Human adipose-derived stem cells (ASCs) are a type of adult stem cell found in adipose (fat) tissue that can be easily isolated from lipoaspirate and cultured for expansion, as they exhibit good proliferation rates and can differentiate into multiple cell types. Indeed, ASCs possess pluripotent capabilities, allowing them to differentiate into various cells, including adipocytes (fat cells), osteoblasts (bone cells), chondrocytes (cartilage cells), and myocytes (muscle cells) [6]. This makes ASCs superior to bone marrow-derived mesenchymal stem cells (BMSCs) for bone regenerative medicine applications [7]. In the presence of ascorbate, glycerophosphate, and dexamethasone, ASCs can differentiate into osteoblast-like cells [8,9]. ASCs hold great promise in bone regeneration, mainly due to their capacity for synthesizing the extracellular matrix and promoting mineralization, which are essential for new bone formation. Different studies underline the advantage of bone grafts based on ASCs combined with biomimetic scaffolds (both natural and synthetic) that protect adult stem cells from mechanical stress, and create the appropriate microenvironment for cell attachment, vitality, and osteogenic differentiation [10,11]. ASC-related osteogenic potential complements the structural support provided by the graft, enhancing the integration and functional repair of bone defects. ASCs combined with bone grafts are used in regenerative therapies for critical-sized bone defects, fractures, and conditions like osteoporosis or non-union fractures [12]. Their ease of harvest and high regenerative potential make them an attractive option for these applications. Moreover, ASCs have a high amount of immunosuppressive properties and can secrete numerous polypeptides, hormones, and effective growth factors, including small non-coding RNAs, to induce osteogenesis. These paracrine effects play a crucial role in therapeutic ASC efficacy, contributing to their ability to enhance tissue repair and modulate the local microenvironment by recruiting endogenous stem cells to the bone defect site [7,13]. ASCs have also been shown to exhibit immunomodulatory properties, suppressing inflammatory responses and promoting tissue regeneration in various disease models through the production of active molecules, such as cytokines and chemokines [14]. This immunomodulatory capacity may be beneficial for reducing inflammation, inhibiting fibrosis, and enhancing tissue healing in the context of bone trauma and defects. Finally, ASCs can be combined with both natural and synthetic scaffolds to support cell delivery, growth, proliferation, migration, and differentiation.

Combinations of these biomaterials along with ASCs and/or growth factors have shown encouraging results for the treatment of bone defects, as demonstrated by both *in vitro* and *in vivo* experiments [15,16]. Indeed, these findings have been translated to several active clinical trials testing the use of ASC–scaffold composites on humans. Preclinical studies have provided compelling evidence supporting the use of ASCs for bone regeneration in various animal models of bone defects and fractures [12]. Therefore, all the characteristics mentioned above make these cells particularly important in clinical use for the treatment of bone loss and defects, including their use in the case of bone grafts. In fact, in the case of an autologous bone graft, ASCs may directly exert their therapeutic action, while in the case of an allogenic bone graft, ASCs may assist the regenerative/healing process, above all thanks to the paracrine anti-inflammatory and immunosuppressive action, thus accelerating bone repair and improving the mechanical properties of regenerated bone. These studies have documented that ASCs can promote new bone development, enhance bone healing, and improve functional outcomes when delivered locally to the site of injury. Moreover, ASCs have been shown to integrate seamlessly into the host tissue, differentiate into osteoblasts, and contribute to the formation of structurally sound bones.

Despite these promising findings, challenges remain in optimizing the delivery, engraftment, and survival of ASCs in the clinical setting. Factors such as cell dosage, delivery vehicle, and patient-specific characteristics (such as age, comorbidities, and the local tissue environment) may influence therapeutic ASC-related outcomes [17]. Determining the optimal number of ASCs required for effective therapy is critical. High doses of ASCs may lead to increased cell death due to nutrient competition or hypoxia at the site of transplantation. Conversely, low doses may be insufficient to achieve significant therapeutic effects. Studies should focus on identifying dose–response relationships and the minimum effective cell number tailored to specific conditions, mainly in critical-sized bone defects or osteochondral diseases. Furthermore, since large-scale production of ASCs is required for many clinical applications, culturing conditions for prolonged *in vitro* expansion of ASCs must be optimized to overcome some critical issues, such as reduced cell proliferation, decreased colony-forming efficiency, and diminished expression of pluripotency markers after extensive passaging [18]. One of the major limitations is also linked to the tendency of ASCs to become senescent and lose their potency for proliferation and differentiation when cultured *in vitro* over time. Therefore, maintaining and/or improving the therapeutic efficacy of ASCs during *in vitro* expansion has become a relevant issue to address. Attempts have been made to enhance ASC stemness by inducing the expression of genes like NANOG or SOX2, but gene transfection poses significant safety risks for clinical applications [19]. Alternatively, FGF2 stimulation has been explored, which boosts initial expansion but restricts long-term proliferation [20]. By addressing these factors, researchers can enhance the clinical translation of ASC-based therapies, paving the way for more effective and reliable treatments for bone defects and other regenerative applications.

Epigenetic alterations have been demonstrated to play a key role in different cellular and molecular pathways. Therefore, the use of epigenetic drugs, able to modulate the actions of epigenetic regulatory factors, control chromatin structure, and regulate gene expression, appears to provide a valuable tool in clinical applications, including regenerative medicine. Several epigenetic molecules, also known as epi-drugs, have been studied for their ability to modulate the activity of epigenetic enzymes such as Histone deacetylases (HDACs) and DNA methyltransferases (DNMTs). Some epi-drugs act as nucleoside analogs, ultimately impairing the activity of such enzymes and modulating the differentiation capacity of ASCs [21], while others act directly on the active binding sites of epigenetic enzymes, thus inhibiting their activity.

RG108 [2-(1,3-dioxoisoindolin-2-yl)-3-(1H-indol-3-yl) propanoic acid or N-Phthalyl-L-tryptofan] is a small molecule inhibitor of DNMT enzymes, DNMT1, DNMT3A, and DNMT3B, which are specifically involved in the process of DNA methylation [22]. This epigenetic mechanism regulates gene expression by adding a methyl group to DNA, thus influencing gene activity without changing the underlying DNA sequence. RG108 works by blocking the activity of the different DNMTs, reducing global DNA methylation content and thereby potentially altering gene expression patterns. Notably, RG108 presents a safe profile and potential absence of off-target effects, even at high concentrations and prolonged exposure [23,24]. Indeed, it has been used in several studies to understand the molecular mechanisms related to decreased DNA methylation in both physiological and pathological conditions [23,24].

In the present study, we aimed to gain deep insight into the metabolic molecular pathways associated with ASC differentiation toward the osteogenic phenotype and to assess the impact of RG108 treatment on osteogenesis. Using NanoString nCounter technology, a powerful technique for multiplexed gene expression analysis without the need for amplification steps, we performed a comprehensive analysis of differentially regulated messenger RNAs (mRNAs) involved in the key metabolic pathways, comparing undifferentiated ASCs with fully osteogenic differentiated ASCs at various time points of induction. *In vitro* validation by quantitative real-time polymerase chain reaction (qRT-PCR) and *in silico* pathway annotation and Gene Ontology (GO) enrichment analysis allowed us to identify specific genes and signaling pathways involved in bone formation and dynamically affected by the inhibition of DNMT activity.

This study comprehensively characterizes the modulation of metabolic pathways occurring during the osteogenic differentiation of ASCs. Moreover, it highlights the influence of epigenetic drugs in enhancing the osteogenic potential of ASCs, thereby offering a novel perspective on their therapeutic application for treating bone lesions and promoting bone regeneration.

## 2. Materials and Methods

### 2.1. Cell Isolation and Culture

Lipoaspirates were obtained after informed consent from three healthy donors (females; age range 55–65 years; BMI < 30 kg/m^2^) undergoing elective liposuction surgery at Policlinico Umberto I, Rome, Italy. Tissue collection was performed in accordance with the principles outlined in the Declaration of Helsinki, and was approved by the Institutional Review Board of the Department of Experimental Medicine of Sapienza University of Rome. Written consent was obtained from all participants. ASC cells were isolated as previously described [25]. Briefly, lipoaspirates were collected and processed under sterile conditions within 24 h. The tissue was washed with sterile phosphate-buffered saline (PBS; Aurogene, Rome, Italy) containing 2% penicillin/streptomycin, then manually minced. The extracellular matrix was digested using a 0.075% Type I collagenase (Gibco, Paisley, UK) solution for 30–60 min in a 5% CO_2_ incubator at 37 °C in a humidified atmosphere. The resulting suspension was filtered through a 100 µm mesh to remove debris, then centrifuged at 2000 rpm for 5 min. The pellets containing the stromal vascular fraction (SVF) were washed with PBS containing 2% penicillin/streptomycin, resuspended in the culture medium, and transferred to a T75 culture flask coated with Type IV collagen (Sigma-Aldrich, Milan, Italy), referred to as passage 0 (P0). ASCs were cultured in DMEM-Ham’s F-12 medium (vol/vol, 1:1) (DMEM/F12; Gibco) supplemented with 10% FBS, 100 U/mL penicillin, 100 mg/mL streptomycin, and 2 mM L-glutamine, and maintained in a 5% CO_2_ incubator at 37 °C in a humidified atmosphere, with medium change twice a week. Cells were detached with 0.5 mM EDTA/0.05% trypsin (Euroclone, Milan, Italy) for 5 min at 37 °C and then replated when they reached 80–90% confluence. Cell morphology was assessed by phase contrast microscopy. All experiments were conducted between passage 3 and 7.

### 2.2. Multichromatic Flow Cytometry (MFC)

To enable optimal simultaneous analysis, the markers were assorted into two panels, one entailing CD34 (Cat. N° 56-0349-42), CD73 (Cat. N° 12-0739-42), and CD90 (Cat. N° 17-0909-42), while the second included CD45 (Cat. N° 130-113-558) and CD105 (Cat. N° 64-1057-42) by CytoFlex (Beckman Coulter). Cell samples were incubated with the specific antibodies in the dark for 30 min at 4 °C, followed by centrifugation at 1200 rpm for 7 min at 4 °C. The pellets were resuspended in PBS containing 10% FBS and then analyzed. Cells were stained with 7-AAD (BD Biosciences, Franklin Lakes, NJ, USA) to differentiate between live and dead populations.

### 2.3. Cell Treatment with RG108 and Proliferation Assay

RG108, a pan-DNMT inhibitor, was purchased from Selleckchem (Suffolk, UK) as lyophilized powder and was reconstituted in DMSO to a final concentration of 10 mM. For RG108 treatment, the culture medium was replaced with a freshly prepared medium containing 50 μM RG108. The RG108-containing culture medium was refreshed every other day. DMSO alone was used as a control at 0.1% (*v*/*v*) concentration.

Proliferation of ASCs treated with RG108 or not was evaluated by the trypan blue dye exclusion method, employing the Countess II Automated Cell Counter (Invitrogen, Carlsbad, CA, USA), according to the manufacturer’s instructions, in three independent experiments.

### 2.4. Osteogenic Differentiation Induction and Evaluation by Alizarin Red-S Staining

Once cells reached 50–60% confluence, they were cultured in osteogenic differentiation medium StemXVivo^®^ Osteogenic/Adipogenic Base Media (R&D Systems, Minneapolis, MN, USA) with RG108 or DMSO for 24 h (T0 point) before adding the StemXVivo^®^ Human Osteogenic Supplement (R&D Systems) for 7 (T7 time point), 14 (T14 time point), and 21 (T21 time point) days to induce osteogenesis. During differentiation, cells were treated with DMSO or 50 µM RG108 every two days. To assess the morphological changes in ASCs treated with RG108 and then induced to osteogenic differentiation, cells were stained by Alizarin Red-S (Merck, Darmstadt, Germany). Briefly, ASCs, treated with osteogenic medium for 14 and 21 days were fixed in 4% paraformaldehyde for 15 min at room temperature (RT) and stained with 2% Alizarin Red-S (Sigma-Aldrich) for 15 min at RT. Cells in four random fields for treatment were photographed at 20× and 40× magnifications using the EVOS XL Core Imaging System (Thermo Fisher Scientific, Waltham, MA, USA) and analyzed using ImageJ 1.5 software.

### 2.5. RNA Extraction and Quantitative Real Time PCR (qRT-PCR)

Total RNA was extracted from ASC cells at baseline (T0) and at time points 7, 14, and 21 days of osteogenic differentiation, and treated with either RG108 or DMSO. RNA isolation was performed by using the Total RNA Purification kit (Norgen Biotek, Thorold, ON, Canada) according to the manufacturer’s instructions. RNA concentration and purity were determined using a Nanodrop ND-1000 Spectrophotometer (Nanodrop Technologies, Wilmington, DE, USA). cDNA was synthesized and analyzed as previously described [26]. Transcript levels of RUNX2 (Hs01047973_m1), ALPL (Hs01029144_m1), COL1A1 (Hs00164004_m1), AOX1 (Hs00154079_m1), c-MYC (Hs00153408_m1), ADH1A (Hs00605167_g1), LAMB1 (Hs01055960_m1), BRCA1 (Hs01556193_m1), BRCA2 (Hs00609073_m1), EZH2 (Hs00544830_m1), and RAD51 (Hs00947967_m1) genes were quantified using specific TaqMan Real-Time Gene Expression Assays (Applied Biosystems, Foster City, CA, USA). All samples were normalized according to GAPDH transcript levels. The relative mRNA levels were analyzed on a QuantStudio 1 Real-Time PCR System (Thermo Fisher Scientific). Gene expression related to redox homeostasis was assessed using SYBR green RT-qPCR, with primers for SOD2 (Fw 5′-TGCACTGAAGTTCAATGGTGG-3′; Rev 5′-CTTTCCAGCAACTCCCCTTTG-3′) and GPX4 (Fw 5′-AGACCGAAGTAAACTACACTCAGC-3′; Rev 5′-CGGCGAACTCTTTGATCTCT-3′). Samples were run on a QuantStudio 1 Real-Time PCR System (Thermo Fisher Scientific). Data were analyzed using the 2^−Δ∆CT^ comparative method [27], and the results are presented as fold change relative to proliferative control cells. Each sample was run in triplicate in at least three independent experiments.

### 2.6. Protein Extracts and Western Blot Analysis

Cells were lysed in RIPA buffer, and Western blot analysis was performed as previously described [28]. Briefly, total proteins (20–100 μg) were resolved under reducing conditions by 7–15% SDS-PAGE and transferred to Immobilon-FL membranes (Millipore, Billerica, MA, USA). The membranes were incubated overnight at 4 °C with primary antibodies to ALPL (Santa Cruz Biotechnology Cat. N°. sc-365765; 1:200 dilution). Primary antibody was followed by the appropriate horseradish peroxidase (HRP)-conjugated anti-rabbit (1:10,000 dilution; Advansta, San Jose, CA, USA) and anti-mouse (1:10,000 dilution; Jackson ImmunoResearch, West Grove, PA, USA) secondary antibody. Hsp90 (Cat. N°. 13171-1-AP; 1:3000 dilution; Proteintech, Rosemont, IL, USA) was used as the internal control. Bound antibody was detected using the WesternBright ECL HRP substrate kit (Advansta) according to the manufacturer’s instructions. Densitometric analysis was performed using ImageJ 1.5 software and band intensities were shown as the fold changed to the corresponding control [29].

### 2.7. Gene Expression Analysis by the NanoString nCounter System

Gene expression analysis was conducted on a set of six total RNA samples derived from two different donors using the NanoString nCounter System and the nCounter Human Metabolic Pathways Panel (NanoString Technologies, Seattle, WA, USA). This panel contains probes for 768 genes involved in over 30 metabolic pathways and 20 internal reference genes for data normalization. Briefly, 100 ng of RNA was hybridized with capture and reporter probe sets (NanoString Technologies) and in the presence of hybridization buffer (NanoString Technologies) at 65 °C for 18 h, according to the nCounter XT CodeSet Gene Expression Assays Protocol (NanoString Technologies). After hybridization, the samples were processed in the nCounter Prep Station (NanoString Technologies) as per the manufacturer’s recommendations, and the data were automatically acquired with the nCounter Digital Analyzer (NanoString Technologies). RNA samples consisted, over the undifferentiated T0 sample, of ASCs at 7, 14, and 21 days after osteogenic differentiation induction, with or without RG108 treatment. The nSolver Analysis Software 4.0 (NanoString Technologies) was used for quality control assessment and raw expression data normalization, considering the geometric mean of the negative control spike-in genes, internal positive controls, and housekeeping genes that encompassed a %CV lower than 30% across all samples. Differentially expressed genes (DEGs) were identified based on a log2 fold change threshold of >1 or <−1, comparing undifferentiated with differentiated ASCs, treated with RG108 or with vehicle (DMSO). A clustered heatmap was generated using the ggplot2 package in R (version 4.2.3) to visualize the expression patterns of key genes involved in metabolic pathways under various experimental conditions relative to the T0 baseline.

### 2.8. Gene Ontology Enrichment Analysis

The functional annotation of the differentially expressed mRNAs at different times of ASC differentiation and RG108 treatment was performed using the Gene Set Enrichment Analysis tool (GSEA, https://www.gsea-msigdb.org/gsea/index.jsp, accessed on 18 November 2024) using the Reactome dataset. Only classes with FDR  ≤  0.05 were considered to indicate significant enrichment and were plotted as river bubble plots using SRplot (https://www.bioinformatics.com.cn/srplot, accessed on 18 November 2024). In addition, an enrichment analysis using Metascape [30]; v3.5.20240901, https://metascape.org/, accessed on 22 November 2024] was conducted to compare RG108-treated cells with vehicle (DMSO)-treated cells. Differentially expressed genes were analyzed by separately processing upregulated and downregulated gene lists at distinct time points using the following ontological sources: KEGG Pathway, GO Biological Processes, Reactome Gene Sets, Canonical Pathways, CORUM, WikiPathways, and PANTHER Pathway. All genes in the genome were used as the background for enrichment. Terms with a *p*-value < 0.01, a minimum count of 3, and an enrichment factor > 1.5 were collected, and selected enriched pathways were plotted as graph bars using Python.

### 2.9. DNA Methylation Data Analysis

DNA methylation profiling data by array (DNAme array) from 4 different subcutaneous adipose tissues were retrieved as BED files from ENCODE [31] under the accession codes ENCSR418YFM, ENCSR315CVG, ENCSR733HHJ, and ENCSR962JMK. BED files were converted in BEDGRAPH using the Bedtools package in R (version 4.2.3) and visualized in Integrated Genome Viewer (IGV, version 2.5.3) using the hg38 human genome. DNA methylation data are reported as methylation scores in a range from 0 to 1, where 1 indicates a fully methylated site while 0 indicates a fully demethylated site.

### 2.10. Statistical Analysis

Data are presented as means ± SD. Statistical analyses were performed using two-way ANOVA or unpaired Student’s *t*-tests, with significance set at * *p* < 0.05, ** *p* < 0.01, and *** *p* < 0.001. Tukey’s correction was applied for multiple comparisons. Data were analyzed on Prism 8.0 (GraphPad Software, La Jolla, CA, USA). All the experiments were conducted in triplicates and repeated at least three times unless stated otherwise.

## 3. Results

### 3.1. Phenotypic Characterization of ASC Cultures and Osteogenic Differentiation Induction Under RG108 Treatment

Recent studies have suggested that RG108 may enhance the efficacy of human MSCs in stem cell therapy [24]. ASCs, isolated from adipose tissue, were phenotypically characterized using flow cytometry with the mesenchymal stem cells markers CD105, CD90, and CD73, and the hematopoietic cells markers CD45 and CD34. FACS analysis showed that 99.46%, 96.44%, and 97.82% of ASCs were positive for CD105, CD90, and CD73, respectively, confirming their mesenchymal identity. In contrast, only 1.90% and 14.47% of cells were positive for the expression of the hematopoietic markers CD45 and CD34, respectively. Treatment with RG108, a non-nucleoside inhibitor that blocks the active site of all the DNMTs, did not affect the stemness characteristics of ASCs, as CD105, CD90, and CD73 levels remained at 99.16%, 94.45%, and 96.68%, respectively. CD45 and CD34 markers were detected in only 1.83% and 15.61% of live cells, respectively.

**Figure 1:**
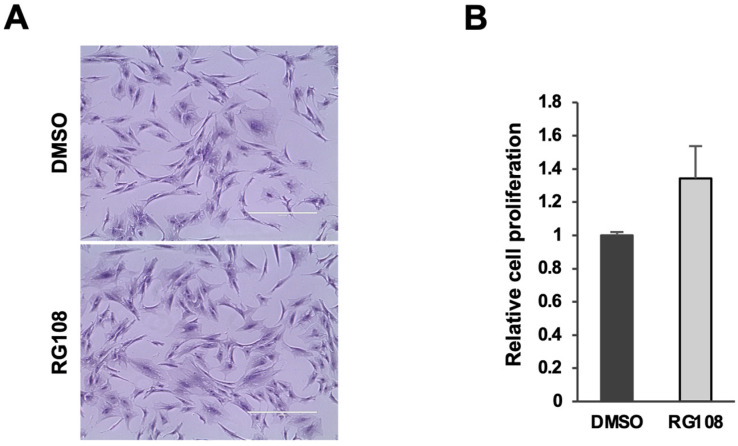
RG108 treatment in ASC morphology and proliferation. (**A**) Phase-contrast microphotographs showing ASCs treated with RG108 or DMSO (as mocked control) for 72 h in culturing medium. Scale bars represent 400 μm. Representative images of three (n = 3) independent experiments. (**B**) Proliferation of ASCs treated with RG108 or DMSO for 72 h using Trypan blue exclusion dye. Bars are median values of three (n = 3) independent experiments.

Additionally, RG108 treatment did not induce any toxicity, as no alterations in cell morphology or proliferative capacity were observed. To assess the impact of RG108 on ASC viability and proliferation, cells were treated with 50 µM RG108 or DMSO (as a control) for 72 h. Cell density and morphology were observed via phase contrast microscopy, and proliferation rates were measured using the Trypan blue exclusion test. No significant changes in cell morphology were noted, and the proliferation rates between RG108-treated and DMSO-treated cells remained statistically comparable (RG108/DMSO = 1.34 ± 0.19; *p*-value not significant) (Figure 1).

**Figure 2:**
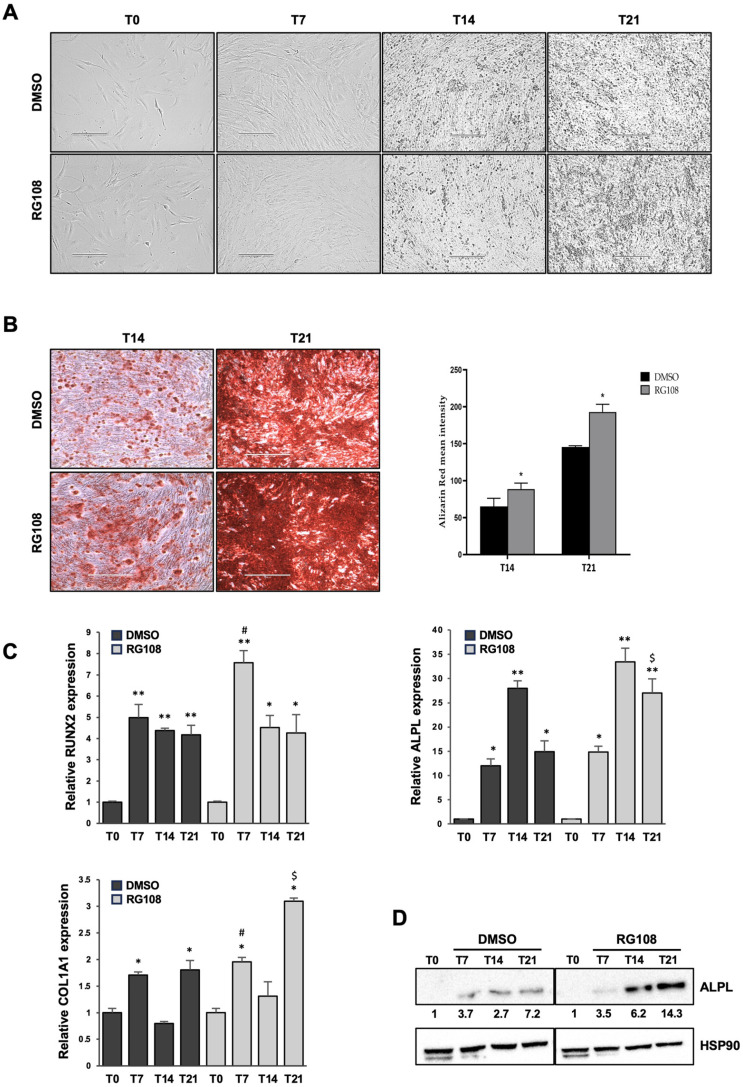
RG108 positively affects ASC osteogenic differentiation. (**A**) Phase-contrast microphotographs showing ASCs treated with RG108 or DMSO (as mocked control) at days 0, 7, 14, and 21 after osteogenic induction. Scale bars: 200 μm. (**B**) Phase-contrast microphotographs depicting mineralization (intense red clusters) in ASCs treated with RG108 or DMSO and stained with Alizarin Red at 14- and 21-day time points of osteogenic induction. Scale bars: 400 μm. (**C**) mRNA expression of osteogenic markers RUNX2, ALPL, and COL1A1 was evaluated by qRT-PCR. Data were normalized to GAPDH mRNA expression. Bars represent means ± SD of three (n = 3) independent experiments, each performed in triplicate. * *p* < 0.05 and ** *p* < 0.01 vs. T0 control; ^#^ *p* < 0.05 vs. T7 DMSO; ^$^ *p* < 0.05 vs. T21 DMSO. (**D**) Western blot analysis of ALPL protein expression in ASCs induced toward osteogenesis with or without RG108 treatment. Hsp90 was used as internal control. Densitometric analysis of ALPL expression is shown as relative expression compared to the T0 control sample.

In order to evaluate the effects of RG108 on ASCs’ capacity to differentiate into osteogenic cells, ASCs were cultured in Osteogenic Differentiation Medium for 7, 14, and 21 days (Figure 2A), while treated or not with 50 μM RG108. Differentiation was assessed by measuring the mineral matrix deposition in fully osteogenically committed ASC cultures on days 14 and 21 of induction, using Alizarin Red staining (Figure 2B). RG108-treated cells demonstrated a significant increase in calcium deposits compared to DMSO-treated cells at both 14 and 21 days, as defined by the quantification of Alizarin Red staining (1.36-fold and 1.32-fold increases over DMSO, respectively). ASCs in the control culture medium (T0) displayed no signs of mineralization. We then analyzed the expression of the key regulators of osteogenesis at the molecular level, such as RUNX2, ALPL, and COL1A1, in ASCs treated with or without the RG108 molecule (Figure 2C). In addition, RG108 treatment seemed to increase *RUNX2* expression after only 7 days of induction, consistent with its crucial role in the early stages of osteogenesis. The oscillating trend in the expression of Type I Collagen, a fundamental component of the extracellular matrix in bone tissue, during the osteogenic differentiation of ASCs, could be influenced by several biological factors, including the temporal regulation of gene expression. The COL1A1 initial increase likely reflects the early activation of the osteogenic differentiation program, while the subsequent decrease could be linked to the production of other matrix proteins. Later, as differentiation progresses, other osteogenic signals may reactivate COL1A1 transcription, which is necessary for the correct transition to fully differentiated osteoblasts and for the formation of a mature collagenous matrix by day 21 of osteogenic differentiation [32]. Transcript levels of the ALPL gene were more prominently increased in ASCs induced toward osteogenesis after 14 and 21 days of induction. ALPL protein expression was also evaluated by WB analysis (Figure 2D), and densitometric analysis confirmed that cells were committed to the osteogenic lineage, with upregulation at 14 and 21 days of differentiation under RG108 treatment, as compared to DMSO-treated samples. Interestingly, at 7 days, ALPL protein levels remained similar to those of DMSO-treated cells, potentially suggesting that the effects of this epigenetic drug on this gene required greater involvement of the differentiation machinery, thus displaying its effects in the late phase of differentiation. Overall, these results denote that RG108 exerts pro-osteogenic effects on ASCs committed to bone formation and mineralization.

### 3.2. NanoString Analysis of Gene Expression in Metabolic Pathways

**Figure 3:**
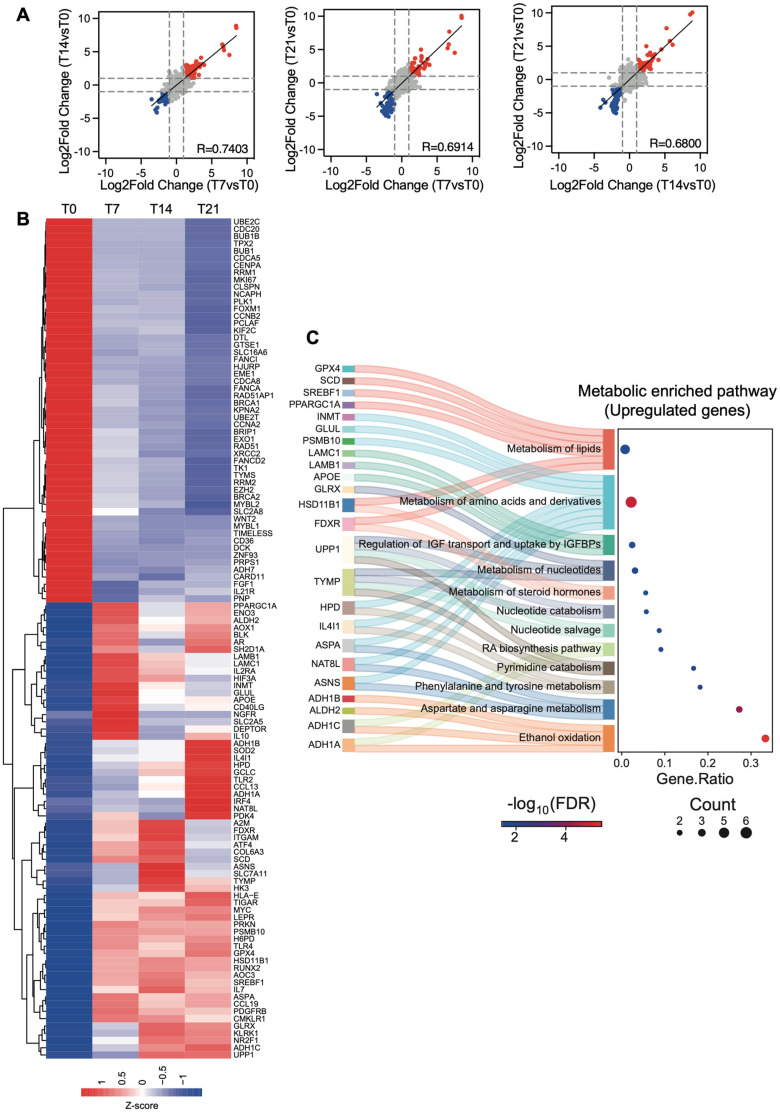
Transcriptional changes during osteogenesis differentiation. (**A**) Correlation analysis of log2 fold changes at reported time points (T7, T14, and T21) compared to T0. Red dots indicate upregulated genes, while blue dots represent downregulated genes. Dashed lines indicate log2 fold change thresholds, set at +1 and −1. (**B**) Cluster heatmap of upregulated and downregulated genes at distinct time points (T0, T7, T14, and T21) identified in (**A**). Expression values are reported as Z-score. (**C**) River plot of metabolic gene set enrichment analysis (REACTOME v2024.1) of upregulated genes. The gene ratio of upregulated genes to total genes in the different pathways is reported. Significance is reported as −log_10_ (FDR) and indicated with color gradient.

Osteogenesis is accompanied by significant metabolic changes, including increased glycolytic activity, enhanced mitochondrial function, and oxidative stress responses, all of which are critical for this type of differentiation. To gain a comprehensive understanding of the expression signatures associated with metabolic reprogramming during the transition from stem cells to osteogenesis, we analyzed transcriptional changes in osteogenically differentiated ASCs at 7, 14, and 21 days using NanoString technology. Specifically, samples were run on the nCounter Metabolic Pathways Panel, which allows for the simultaneous measurement of 768 genes related to metabolism, including those involved in energy production, biosynthesis, catabolism, cell stress, and signal transduction. We integrated gene expression profiling data with bioinformatics analysis, and, through this combined approach, we identified key pathways and pinpointed hub genes that are modulated during osteogenesis. The nSolver Advanced Analysis system revealed a strong correlation between gene expression changes across the time points analyzed, suggesting the establishment of a transcriptomic program in the early phase of differentiation, which is maintained throughout osteogenesis (Figure 3A).

**Figure 4:**
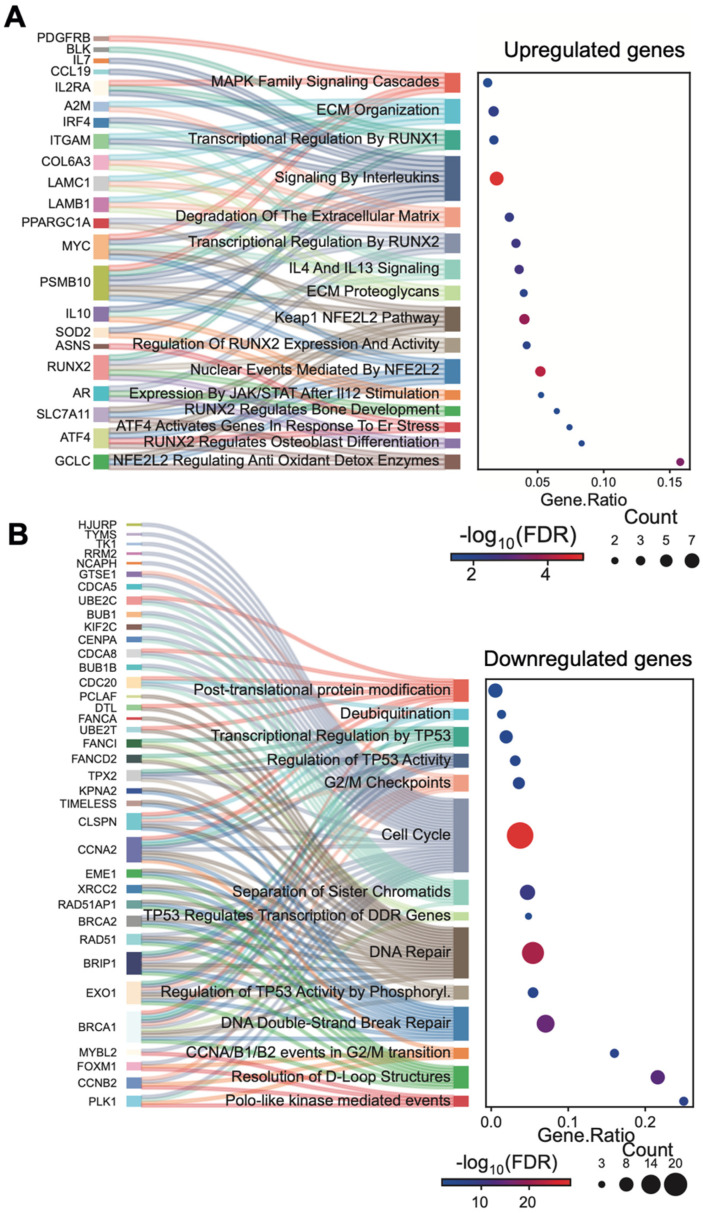
River plots of upregulated genes (**A**) and downregulated (**B**) genes related to non-metabolic processes during ASC osteogenic differentiation.

In detail, we identified 53 downregulated genes and 63 upregulated genes across all the differentiation time points (T7, T14, and T21) analyzed, compared to control ASCs (T0) (Figure 3B). Moreover, we performed gene set enrichment analyses on both upregulated and downregulated transcripts using the Reactome dataset (Figure 3C). Interestingly, we observed significant enrichment in metabolic pathways only for the upregulated genes across the samples, highlighting that osteogenic differentiation is characterized by an increase in metabolic turnover to meet the heightened energy demands necessary for successful differentiation. In particular, we found enrichment in metabolic pathways that are known to be key regulators of osteogenic differentiation, such as amino acid and lipid metabolism [33]. Indeed, lipids and amino acids are essential raw materials for osteoblasts, required for protein synthesis and ATP production. We observed the upregulation of key genes involved in lipid metabolism, including Ferredoxin reductase (FDXR), a target gene of RUNX2 [34]. FDXR is a mitochondrial flavoprotein that initiates electron transport for cytochromes P450 by receiving electrons from NADPH [35], playing a central role in the production of the energy needed for differentiation. Additionally, we identified the upregulation of GPX4, an enzyme that converts lipid hydroperoxides into lipid alcohols, thereby preventing the iron (Fe^2+^)-dependent formation of toxic lipid reactive oxygen species (ROS) [36]. Regarding amino acid metabolism, we found an increase in ASNS, the enzyme responsible for synthesizing asparagine from glutamine and aspartate, which is known to be upregulated during osteoblast differentiation [37]. Altogether, these data confirm that ASCs efficiently activate the metabolic pathways necessary for the osteogenic program. Furthermore, the pathways related to IGF transport and uptake were upregulated, in line with the promotion of glucose absorption, which is subsequently catabolized during glycolysis to provide the ATP required for osteoblast differentiation [38]. In particular, we found that the upregulation of LAMC1, a component of the extracellular matrix, promotes osteogenic differentiation [39], and its expression is induced by IGFBPs [40]. Laminin subunit gamma 1 activates downstream signaling pathways, including Rho GTPase and MAPK, through its interaction with integrins. This interaction regulates cellular processes such as bone morphogenesis and bone matrix deposition, and it also plays a crucial role in the directional differentiation of stem cells into osteoblasts [41]. We also observed the upregulation of nucleotide metabolism, which is known to contribute to osteoclast differentiation. This increase is likely related to high energy demands during differentiation. Notably, we found upregulation of the UPP1 gene, involved in the cleavage of uridine into uracil and ribose-1-phosphate, to be a key component in glycolysis that increases ATP production [42]. Additionally, steroid hormone metabolism, which regulates bone development and homeostasis, was found to be upregulated. Interestingly, among the upregulated genes in this pathway, we identified FDXR, a known target of RUNX2, a master transcription factor for osteogenesis [34]. Although the role of retinoic acid (RA) in osteogenesis remains debated, we observed the induction of genes involved in RA biosynthesis, suggesting a potential pro-osteogenic function in ASCs, as supported by Jacobsen et al. [43] and James et al. [44]. Elevated levels of ethanol (EtOH) are reported to impair bone formation. Among genes involved in EtOH oxidation, a key step in its metabolism, we found increased expression of ALDH2, an enzyme that converts acetaldehyde (a toxic intermediate of EtOH oxidation) into acetate. Moreover, it has been reported that ALDH2 promotes osteogenic differentiation by inhibiting ferroptosis and activating the antioxidant factor NRF2 [45]. Accordingly, among the upregulated genes that are not strictly related to metabolic upregulated pathways (Figure 4A), we observed enrichment in NRF2-regulated processes (e.g., nuclear events mediated by NFE2L2; the KEAP1 and NFE2L2 pathways, and the regulation of antioxidant detoxifying enzymes by NFE2L2).

Notably, pathways associated with matrix formation and organization were also upregulated. Altogether, these changes could influence stem cell fate during osteogenesis, supporting ASC commitment to bone tissue development [46]. Consistent with expectations, we observed enrichment in processes regulated by key osteogenic factors, such as RUNX1 [47], RUNX2 [48], ATF4 [49], MAPKs [50], and JAK-STAT signaling [51] (Figure 4A). On the other hand, in line with previous findings, we observed a notable downregulation of genes involved in cell cycle progression, sister chromatids separation, and DNA damage repair (Figure 4B). To gain further insights into the timing of the differentiation process, we studied the modulation of gene expression at distinct time points within the NanoString annotated pathways (Supplementary Figure S1, Tables S1 and S2). Taking a comprehensive view of the temporal dynamics in the activation of metabolic pathways, we observed the following patterns: (i) increased gene expression primarily during the early phase of differentiation for fatty acid oxidation and tryptophan-kynurenine metabolism; (ii) relatively stable upregulation throughout differentiation for AMPK and glycolysis; (iii) continuous upregulation throughout the entire process with a peak in the advanced or terminal phase for pathways such as reactive oxygen response, MAPK (with NGFR as the most upregulated gene across all differentiation times), TCR, TLR, NFKB, mTOR, and autophagy signaling (Supplementary Figure S1). Moreover, among the most upregulated biological processes, we observed a progressive increase in transcriptional regulation across all differentiation time points. Interestingly, the hypoxia-inducible transcription factor HIF3A was significantly upregulated throughout the entire osteogenic process (Supplementary Figure S1 and Table S1), unlike HIF1A, which remains stable during differentiation. This suggests a possible specific and HIF1-independent role for HIF3A in the osteogenic differentiation of ASCs.

Overall, NanoString data suggest that the metabolic profile of ASCs is dynamically reprogrammed during both the early and late stages of osteogenic commitment. This reprogramming includes the upregulation and downregulation of various genes, all of which are essential for orchestrating and supporting the conditions necessary for the complex commitment toward osteogenic lineage.

### 3.3. NanoString Result Validations with qRT-PCR

**Figure 5:**
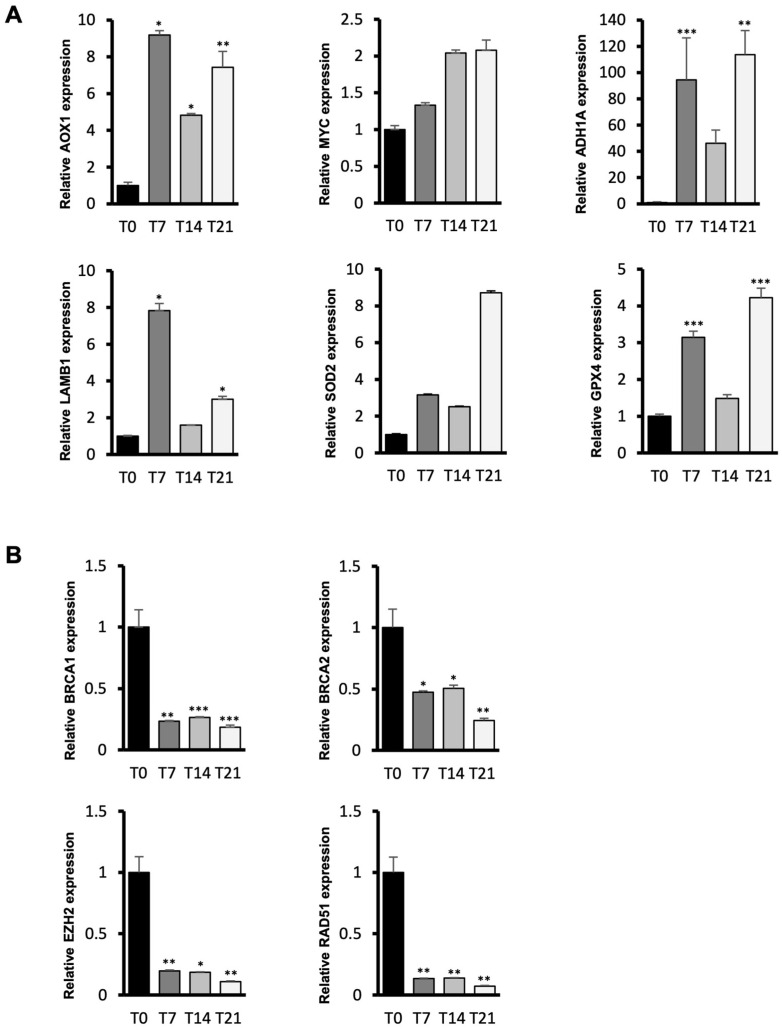
NanoString result validation by qRT-PCR. (**A**) qRT-PCR results of selected upregulated and (**B**) downregulated genes across ASC osteogenic differentiation compared to the T0 control sample. Transcript levels were normalized to GAPDH mRNA expression. Bars represent means ± SD of three (n = 3) independent experiments, each performed in triplicate. * *p* < 0.05; ** *p* < 0.01; *** *p* < 0.001 vs. T0 control sample.

To validate the NanoString results, we confirmed the expression levels of a subset of differentially expressed genes in enriched pathways at different time points of osteogenic induction by performing qPCR experiments (Figure 5).

Among the upregulated genes, we selected AOX1, which has been recently identified as a potential osteogenic marker in human mesenchymal stem cells [52], MYC, which promotes BMP2-dependent osteogenesis in human MSCs [53], ADH1A, which regulates IGF transport and uptake as well as ECM organization, and SOD2 and GPX4, two genes involved in ROS detoxification, a key mechanism for reducing ROS levels produced during differentiation (Figure 5A). AOX1 shows an expression pattern consistent with its role as an osteogenic marker. The robust increase in the early phase (9.1-fold vs. T0) indicates that osteogenic commitment is quickly and successfully initiated, and that this upregulation is maintained throughout the differentiation period, with only a slight physiological decrease in expression levels toward the later stages, which does not hinder the success of differentiation. LAMB1 displays a peak in expression at the early T7 time point (7.8-fold vs. T0), and ECM rearrangement is likely a cellular response to the changes required during the cell commitment phase for osteogenic differentiation. As for the antioxidant genes SOD2 and GPX4, their expression increases throughout the differentiation process, with peaks at the T21 time point (8.7-fold and 4.2-fold vs. T0, respectively) corresponding to the final phase of osteogenic differentiation. This peak in expression aligns the need for antioxidant action with the accumulated ROS production during the earlier phases. Among the downregulated genes, we tested BRCA1, BRCA2, EZH2, and RAD51, all of which are involved in DNA damage repair and expected to be downregulated in non-proliferating cells (Figure 5B). Indeed, these genes exhibit strong downregulation from the early stages of osteogenic commitment, with the most significant reduction observed at the T21 time point (0.18-fold, 0.24-fold, 0.1-fold, and 0.07-fold vs. T0, respectively). This is consistent with the non-proliferative state of the cells in the final stage of osteogenic differentiation.

### 3.4. Effects of RG108 Treatment on Metabolic Pathways During Osteogenic Differentiation of ASCs

**Figure 6:**
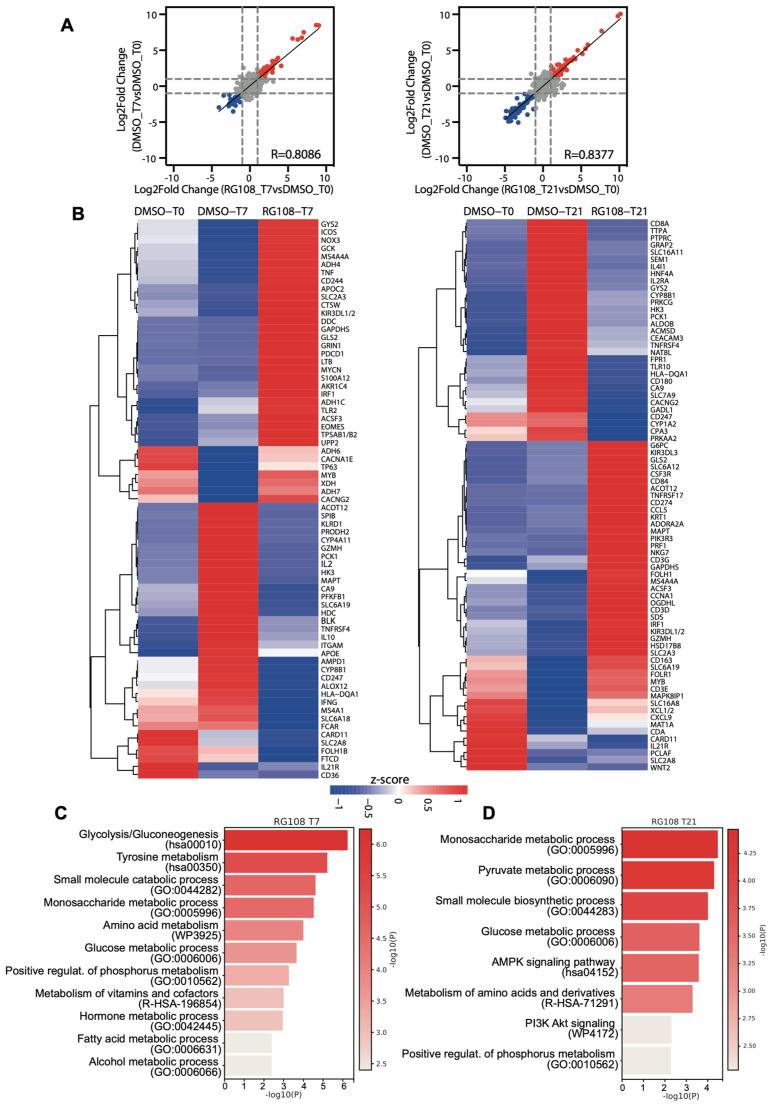
Transcriptional changes during osteogenesis differentiation of ASCs treated with RG108. (**A**) Correlation analysis of log2 fold changes in ASCs treated or not with RG108 at reported time points (T7, T21) compared to T0. Red dots indicate upregulated genes, blue dots indicate downregulated genes. Dotted lines indicate log2 fold change thresholds set at +1 and −1. (**B**) Cluster heatmap of upregulated and downregulated genes after RG108 treatment at distinct time points (T0, T7, and T21) identified in (**A**). Expression values are reported as Z-score. (**C**) Graph bars of enriched pathways for RG108 T7-upregulated genes calculated with Metascape tool. X axis shows significance reported as −log10 *p* value (*p*). (**D**) Graph bars of enriched pathways for RG108 T21-upregulated genes calculated with Metascape tool. X axis shows significance reported as −log10 *p* value (*p*).

We explored the impact of RG108 on ASC osteogenic potential to identify statistically significant changes in specific metabolic processes in the early (RG108 T7) and late (RG108 T21) stages of osteogenic differentiation (Figure 6). The evaluation of global DNA methylation content, performed on total genomic DNA isolated from ASCs treated with or without 50 µM RG108, indicated a significant reduction (approximately 50% in early stages and 30% at T21) in 5-methylcytosine (5mC) levels in the RG108 groups relative to controls.

As shown in Figure 6A, NanoString analysis revealed a strong correlation between the gene expression changes observed in DMSO-treated differentiation and RG108 treatment, suggesting that RG108 does not induce a distinct transcriptional program compared to standard DMSO differentiation, but rather enhances the transcriptional effects observed in Figure 3A. At the T7 time point, we identified 35 upregulated genes and 32 downregulated genes in RG108-treated ASCs compared to DMSO-treated controls. At 21 days of differentiation, 40 genes were upregulated, and 31 genes were downregulated with RG108 treatment relative to DMSO (Figure 6B). Interestingly, at T7, 28 genes were upregulated compared to T0, while 7 genes were downregulated after 7 days of differentiation. Notably, RG108 treatment helped maintain the expression levels of these seven downregulated genes. Among these, TP63, which transcriptionally regulates the TNF receptor superfamily member 11b (TNFRSF11B), promoting bone remodeling and inhibiting osteoclastogenesis [54], and MYB, a transcription factor identified as promoting osteogenesis [55], were among the key genes. Additionally, two genes involved in alcohol oxidation, ADH6 and ADH7, were upregulated. An enrichment pathway analysis performed using Metascape [30] identified several interesting metabolic pathways upregulated at T7 and T21 days of RG108 treatment, compared to standard differentiation (Figure 6C). At T7 we observed increased expression of genes involved in glucose, fatty acid, alcohol, and amino acid metabolism, supporting the hypothesis that RG108 enhances pro-differentiation pathways (Figure 6C). Moreover, the phosphorus metabolic process was upregulated, which has previously been linked to osteocyte maturation [56]. At 21 days of RG108 treatment, in addition to the upregulation of metabolic processes, we identified overexpressed genes in AMPK and PI3K-AKT signaling. The hyperactivation of the AMPK pathway stimulates osteogenesis while inhibiting adipogenesis [57], and the PI3K-AKT axis promotes BMP2-induced osteogenesis [58]. Supplementary Figure S2 and Table S2 display the upregulated and downregulated genes in RG108-treated samples compared to standard differentiation, focusing on Nanostring-annotated pathways at selected differentiation time points. These data indicate that RG108 treatment promotes a stronger activation of amino acid synthesis and transcriptional regulation throughout the entire differentiation process. Additionally, RG108 treatment is associated with increases in glycolysis, MAPK and NFκB signaling, glutamine metabolism, and fatty acid oxidation at the early stages of differentiation, while promoting TCR signaling upregulation in the terminal phase. Notably, in the context of transcriptional regulation, besides the overexpression of the previously noted MYB, RG108 treatment also induces the upregulation of IRF1, with its more pronounced effects at the later stages of differentiation. IRF1 has been implicated in the regulation of osteogenic differentiation in human BMSCs via the lncRNA XIST/miR-450b/FBXW7 axis [59]. Among the downregulated genes, we found enriched pathways related to immune response, allogeneic rejection, and inflammatory response (Supplementary Figure S3). Interestingly, HLA-DQA1, a class II HLA molecule expressed on antigen-presenting cells with a well-established role in the immune system [60], was downregulated and featured in several of the enriched downregulated pathways (Supplementary Figure S3).

**Figure 7:**
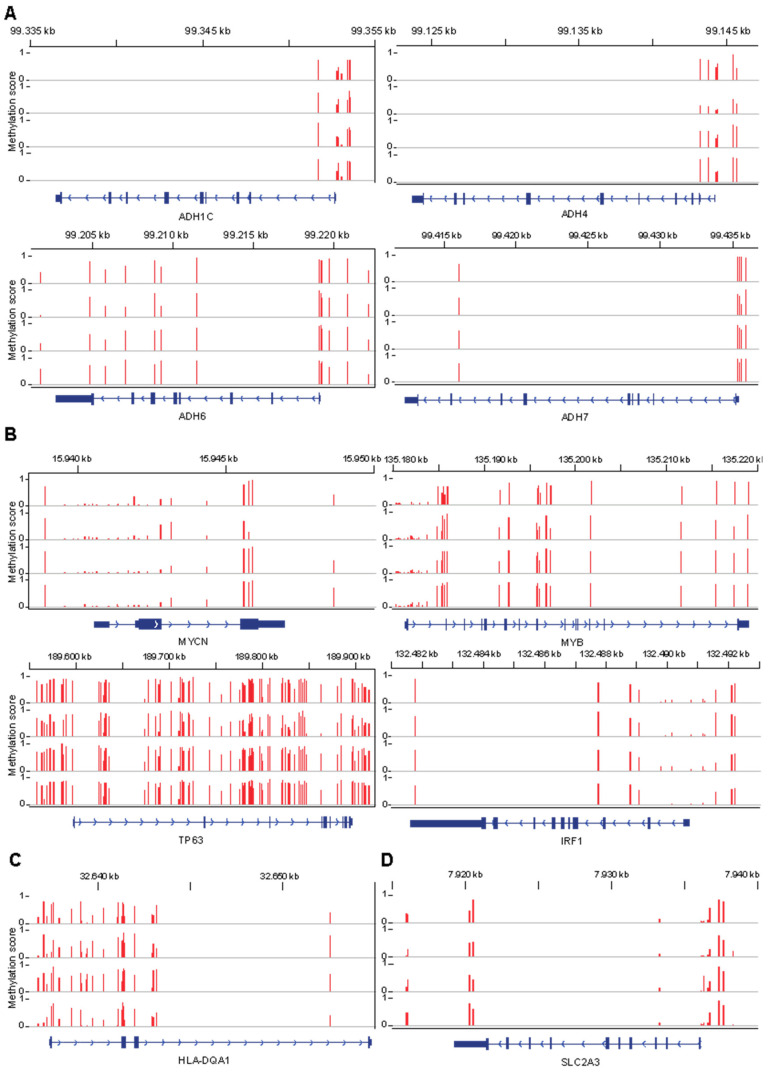
DNA methylation profiles of genes deregulated by RG108 treatment during ASC osteogenesis. (**A**) DNA methylation array data from four different subcutaneous adipose tissues showing methylation status of genes involved in ethanol oxidation (ADH1C, ADH4, ADH6, and ADH7) regulated by RG108. (**B**) DNA methylation status of transcription factors (MYCN, MYB, TP63, and IRF1) whose expression is upregulated by RG108 treatment. (**C**) DNA methylation profile of HLA-DQA1. (**D**) DNA methylation status of SLC2A3. Data are reported as methylation scores in a range from 0 to 1, where 1 indicates a fully methylated site while 0 indicates a fully demethylated site.

Finally, the observed transcriptional differences between RG108- and DMSO-treated ASCs are consistent with the analysis of the DNA methylation array data of four subcutaneous adipose tissue samples from the Encode project [30], which showed that genes involved in EtOH oxidation (Figure 7A), transcription factors (Figure 7B), allogeneic rejection (Figure 7C), and glucose metabolism (Figure 7D) are highly methylated. This suggests that RG108 modulation of DNA methylation may be directly responsible for these transcriptional changes, further supporting its role as a pro-osteogenic compound.

## 4. Discussion

Bone defects and skeletal system diseases have a remarkable impact on patients’ lives and represent a major burden on healthcare systems worldwide. These conditions, arising from trauma, genetic disorders, or degenerative diseases, often result in chronic pain, limited mobility, and decreased quality of life. Addressing these challenges through targeted medical treatments, especially regenerative medicine therapies, is crucial to improving patient outcomes and reducing healthcare costs. Regenerative therapies aim to restore the structure and the function of damaged skeletal tissue by promoting or enhancing the natural healing process. These therapies can offer more effective and long-lasting solutions compared to traditional methods like prosthetics or implants, with the added benefit of minimizing the risk of complications and rejection.

Adipose-derived stem cells (ASCs) have emerged as promising tools in regenerative medicine due to their regenerative potential, multidirectional differentiation capacity, and immunomodulatory properties. Specifically, ASCs have demonstrated significant promise in treating bone defects and trauma. They contribute to bone homeostasis by differentiating into osteogenic lineages and modulating the surrounding cellular environment. Wagner et al. [61] showed that ASCs modulate T cells’ viability, playing a critical role in bone remodeling in osteoporosis patients. ASCs from donors with osteoporosis exhibited superior proliferation, differentiation, and bone regeneration abilities compared to MSCs, highlighting their relevance in maintaining bone homeostasis. Additionally, Freitas et al. [62] found that the quality of bone tissue formed by ASCs is comparable to natural bone in terms of mechanical properties, thus making these adult stem cells the most attractive candidates for long-term regenerative applications in treating bone injuries. Additionally, ASCs have shown clinical effectiveness in improving joint mobility and reducing pain in knee osteoarthritis, with outcomes comparable to traditional therapies, as reported in clinical trials like NCT02674399. Considering their safety and efficacy in bone regeneration, ASCs are a valuable option for cell transplantation therapy, particularly in conditions such as osteoarthritis and fractures. However, as the clinical application of ASCs for systemic diseases is still under investigation, further research is required to optimize *in vitro* expansion, dosage, administration methods, and long-term outcomes. Enhancing specific biological characteristics of ASCs, such as self-renewal, proliferative capacity, and differentiation potential, is essential to maximize their efficacy in bone regeneration.

In the present study, we aimed to gain a deeper insight into the molecular pathways modulated in ASCs induced toward osteogenic differentiation, in order to clarify their functional involvement in this process. Such findings will improve our knowledge of the biology of these stem cells during differentiation and help identify specific processes that can be targeted to enhance ASC efficacy in clinical applications. We specifically focused on analyzing the metabolic events occurring during osteogenic differentiation, because the interplay between metabolic pathways and signaling networks ensures that osteoblast precursors have the necessary resources for proliferation, differentiation, and mineralization. While the molecular details of this interplay are not fully understood, it is well established that the bioenergetic nature of bone cells plays a crucial role in maintaining bone health [63], highlighting the importance of further investigation into this specific aspect.

Using the nCounter NanoString technology, a molecular profiling tool that enables accurate, reproducible, and multiplexed quantification of mRNAs through digital barcoding, thus bypassing the need for amplification, we characterized the key regulatory pathways involved in cellular metabolic processes that are essential for providing the required energy and biosynthetic precursors for ASC osteogenic commitment. Firstly, our analysis confirmed that ASCs may serve as precursors of osteogenic cells, as evidenced by the activation of central osteoblast differentiation pathways, such as amino acid and lipid metabolisms. A key aspect of osteogenic differentiation is glucose metabolism, which provides the primary energetic source required for differentiation. We observed an enrichment in the regulation of IGF transport and uptake, processes known to promote glucose absorption under high ATP demand, which is essential for collagen biosynthesis and mineralization during bone remodeling [64]. Our study found dynamic gene expression changes over time in the early, middle, and late stages of osteogenic differentiation of ASCs, mirroring the fundamental metabolic, molecular, and transcriptional adaptations needed to support the differentiation process and the maturation of osteocytes. In particular, the gene expression shifts from osteogenic precursors to osteocytes could reflect the metabolic transition from a proliferative phase (sustained by glycolysis, fatty acid oxidation, amino acid metabolism, immune response modulation, and inflammatory regulation) to a differentiation phase (oxidative metabolism through AMPK activation and synthesis of extracellular components as bone matrix and structural proteins through protein synthesis) supported by transcriptional regulation activation, to a late phase, which enhances cellular maturation and bone turnover (MAPK and NF-kB pathways), mineralization (TCR and TLR signaling and ROS response), and bone homeostasis (autophagy). Of particular interest among the enriched metabolic pathways, also due to the high levels of upregulation of the involved genes compared to the average, is ethanol (EtOH) oxidation, which has not yet been deeply studied in osteogenesis. The marked upregulation of the ADH1A, ADH1B, ADH1C, and ALDH2 genes during osteogenesis could regulate the redox equilibrium and cellular homeostasis in osteoblasts. Indeed, EtOH metabolism contributes to the NAD⁺/NADH balance, which is crucial for various cellular functions, including energy production. Maintaining an adequate redox balance in osteoblasts is especially vital for their proper proliferation and differentiation. An imbalance in NADH accumulation could impair bone mineralization, whereas the recycling of NAD⁺ supports bioenergetic processes necessary for differentiation. The generation of metabolites like acetate, which can be converted to ATP in the Krebs cycle, further supports the energy demands of bone formation and growth. Proper alcohol metabolism may also contribute to regulating local inflammation and maintaining the balance between bone formation and resorption, potentially influencing the equilibrium between osteoclasts and osteoblasts. Furthermore, being involved in collagen synthesis, the upregulation of enzymes involved in EtOH metabolism might have indirect effects on bone matrix. For instance, collagenase, an enzyme implicated in collagen degradation, could be partially regulated by the redox balance influenced by EtOH metabolism. The activity of ADH and ALDH enzymes is also important in controlling redox activity, by mitigating cellular damage caused by reactive oxygen species (ROS). On the other hand, a properly balanced level of ROS expression is central, since these species could also serve as signaling molecules with a positive role in the mineralization process, bone remodeling, and osteoblast differentiation [64]. In this scenario, the strong upregulation of AOX1, an enzyme involved in the oxidation of aldehydes and nitrogen-containing compounds, adds another layer of understanding to the metabolic adaptations during osteogenesis. AOX1 and ADH1 work together to control ROS levels, which can influence differentiation through downstream signaling cascades.

By managing cellular stress and the detoxification of harmful aldehydes, AOX1 additionally modulates the availability of metabolic intermediates necessary for ASCs to differentiate into osteoblasts. Specifically, the observed upregulation of AOX1, ADH1, and SOD2 genes appears to play a coordinated role in ensuring a stable redox environment and efficient bioenergetic support during the biological processes essential for bone formation. This also occurs during osteogenic differentiation of human mesenchymal stem cells, where the synergic regulation of mitochondrial biogenesis and the finely tuned action of antioxidant enzymes help to limit the excess of ROS that are physiologically produced in a metabolically active condition such as cellular differentiation [65]. This precise regulation of ROS might be coordinated or supported by the transcription factor HIF3A, which is highly upregulated throughout the differentiation process. Recent studies have shown that this third alpha-class hypoxia inducible factor subunit can modulate oxidative stress and mitigate ROS-induced damage under hypoxic conditions in chronic obstructive pulmonary disease, by influencing pathways involved in redox biology, such as the activation of GPX4 [66]. It is reasonable to speculate that the HIF3A upregulation could support the formation and maturation of osteocytes under hypoxic conditions. Furthermore, HIF3A may function similarly to HIF1, regulating the expression of specific osteogenic genes [67]. Future studies could clarify this aspect and further investigate the potential role of HIF3A in osteogenesis.

Interestingly, the ADH1 enzyme is also involved in the metabolism of retinol to retinal, a precursor to retinoic acid (RA), which is a powerful regulator of various cell differentiation processes, including osteogenesis. Recent evidence suggests that ADH1 influences the composition of the extracellular matrix (ECM), a critical factor during osteogenic differentiation improving the structural integrity of the microenvironment of differentiated cells. Similarly, the LAMB1 gene, which encodes for the laminin beta 1—a subunit of laminin that is a key ECM component—plays an important role in osteogenesis by providing structural support and biochemical signals that correctly influence cell fate. In particular, it regulates cell attachment to the matrix, a pivotal factor in the differentiation process. Although LAMB1 is not a direct marker of osteogenesis, it may contribute to osteogenic differentiation by modulating the ECM environment, thereby potentially affecting cell adhesion and signaling pathways essential for osteoblast maturation and function. Moreover, laminin interactions with integrins (such as integrin β1) activate pathways like MAPK/ERK and PI3K/AKT, which are important for osteoblast differentiation and mineralization. Adding to this regulatory network, the nerve growth factor receptor (NGFR) has recently been identified as a key player in osteogenesis and bone formation, by controlling several pathways, including AKT and MAPK, and by exerting an epigenetic activating role, as demonstrated by recent studies [68]. Our findings further support this role, as NGFR is one of the most prominently upregulated genes at T7 during osteogenic differentiation.

Moreover, we aimed to test the effect of the epigenetic drug RG108 on this differentiation process. Indeed, this pan-DNMT inhibitor has been shown to enhance the osteogenic program in periodontal ligament cells by increasing RUNX2 expression and nuclear localization, accelerating the expression of osteogenic markers and promoting mineralization [69]. Furthermore, unlike other epigenetic drugs such as 5-aza [21], RG108 is known to exert positive effects on cellular senescence [70,71]. The evaluation of mineral matrix deposition via Alizarin Red Staining confirmed a robust increase in calcium deposits, commonly associated with terminal osteogenic differentiation in ASCs subjected to RG108 treatment, compared to the mock-treated DMSO control. This clearly indicates an increased presence and depository activity of osteogenically differentiated cells. In agreement with this, critical determinants of osteogenic differentiation, such as RUNX2 and ALPL, showed elevated levels of expression, suggesting an enhancement of osteogenic commitment upon RG108 treatment and confirming the role of DNA methylation in regulating the transcriptional activation of genes with a pivotal role in the osteogenic differentiation of ASCs [72].

Transcriptional changes in metabolic profiles suggest that treatment with RG108 could better support the metabolic demands during ASC differentiation, potentially promoting a transition toward a more energy-favorable metabolism to support both proliferation and the early stages of the differentiation. This includes the enhanced metabolism of glucose, fatty acids, alcohols, and amino acids, as well as the activation of alternative energy sources to sustain matrix formation and mineralization (such as the phosphorus metabolic process). Moreover, RG108 seems to modulate the transcriptional activity of differentiating cells in a pro-osteogenic direction, upregulating transcription factors like MYB and IRF1, while sustaining the expression level of TP63, thereby favoring osteogenesis and bone remodeling. Furthermore, RG108 likely acts as a modulator of specific pro-differentiation signals, enhancing the activation of pro-osteogenic pathways like AMPK and PI3K-AKT, while decreasing the immune response through the downmodulation of genes involved in the inflammatory response and allogeneic rejection. This may potentially promote a more favorable microenvironment for osteocyte maturation.

Our results suggest that RG108 can enhance osteogenic differentiation without disrupting the pathways essential for cellular proliferation and commitment. This is particularly reassuring as it indicates that, unlike similar compounds, RG108 can modulate DNA methylation and drive key cellular changes without significantly affecting cell viability or unbalancing the overall differentiation process. Thus, it is able to induce changes with fewer side effects, even with prolonged exposure, and maintain the regenerative potential of stem cells over time [22,73]. In agreement with these results, in RG108-treated ASCs, we identified increased levels of RUNX2 and ALPL, both critical determinants of osteogenic differentiation. Specifically, the ALPL gene, by facilitating hydroxyapatite deposition, plays a crucial role in bone formation. Its expression levels showed a dynamic upregulation upon RG108 treatment, with a peak occurring around day 14 of differentiation and reaching maximum expression at day 21, marking osteoblast maturation. Interestingly, ALPL upregulation and activity appear to correlate with the high capacity of human mesenchymal stem cells to form bone *in vivo* [74]. On the other hand, the RUNX2 gene, which encodes a transcription factor that acts as an early regulatory switch driving ASCs toward the osteogenic pathway, shows an increased expression shortly after osteogenic induction, with more pronounced differences between RG108- and DMSO-treated cells at day 7 of differentiation. These data confirm the role of DNA methylation in controlling the transcriptional activation of different genes, including RUNX2 and ALPL [72], and offer the potential to use a less toxic and more stable epigenetic drug to improve ASC multipotency. Indeed, RG108 is known for its non-cytotoxic nature, and our study suggests that it represents a more preferable option compared to other DNA-demethylating agents, such as 5-azacytidine and its derivatives, for inducing osteogenic differentiation in ASCs by upregulating key osteogenic genes without significant cytotoxicity or genomic instability. RG108 modulates DNA methylation without significantly impacting cell viability, even with prolonged exposure. Its low toxicity can be partly attributed to its mechanism of action, which involves direct binding to the catalytic site of DNMTs rather than integration into the genome [22,73,75]. This property suggests fewer side effects during clinical applications, especially in regenerative medicine, where minimizing adverse effects is critical. Our data are in agreement with recent evidence emphasizing the role of epigenetic mechanisms, particularly DNA methylation, in regulating gene expression during ASC differentiation. RG108 treatment enhances the transcriptional and metabolic landscapes conducive to osteogenic differentiation while simultaneously reducing inflammatory and immune-related pathways.

These findings highlight the potential of RG108 as a valuable adjuvant in regenerative medicine strategies targeting bone repair and remodeling. Indeed, RG108 may be an effective epigenetic drug to be introduced in future applications using ASC-based therapies for bone regeneration. Moreover, leveraging epigenetic modulation could pave the way for developing more targeted and personalized therapies for skeletal disorders. Future experiments will be focused on fine-tuning the clinical application of ASCs by integrating innovative natural or synthetic biomaterials and also conducting *in vivo* studies to evaluate the durability of bone regeneration and the risk of potential side effects (e.g., tumorigenicity), before optimizing protocols and translating these cell-based therapies into clinical practice.

## 5. Conclusions

The present study reveals the intricate molecular and metabolic dynamics involved in the osteogenic differentiation of ASCs, with a particular emphasis on the influence of the pan-DNMT inhibitor RG108. This epigenetic drug not only enhances osteogenesis-related markers, such as RUNX2 and ALPL, but also modulates key metabolic and transcriptional pathways, including ethanol oxidation, glucose metabolism, and transcriptional regulation. The identification of potential osteogenic drivers, such as AOX1 and ADH1A, further expands our understanding of the mechanisms governing ASC biology. These results underscore the potential of RG108 as a promising epigenetic modulator to optimize ASCs for regenerative applications, paving the way for more effective and personalized cell-based therapies in bone regeneration.

## Data Availability

The original contributions presented in this study are included in the article/Supplementary Material. Further inquiries can be directed to the corresponding author.

## Supplementary Materials

The following supporting information can be downloaded at: https://www.mdpi.com/article/10.3390/cells14020135/s1, Supplementary Figure S1. NanoString pathways annotation of modulated genes during ASC osteogenic differentiation. Bar plots showing modulated genes within NanoString annotated pathways at T7 (A), T14 (B), and T21 (C) compared to T0. Every square represents a single gene. Pathways were reported when at least 3 genes were modulated. Color bars show downmodulated (blue) and upregulated (red) genes. Supplementary Figure S2. NanoString pathways annotation of modulated genes during RG108 treatment compared to standard ASC osteogenic differentiation. Bar plots showing modulated genes within NanoString annotated pathways at T7 (A) and T21 (B) compared to T0. Every square represents a single gene. Pathways were reported when at least 3 genes were modulated. Color bars show downmodulated (blue) and upregulated (red) genes, reported as log2 fold change. Supplementary Figure S3. Transcriptional changes during osteogenesis differentiation of ASCs treated with RG108. River plots of upregulated genes at T7 (A) and at T21 (B), and downregulated genes at T7 (C) and T21 (D) of RG108 treatment compared to DMSO-treated cells. Graph bars of enriched pathways for RG108 T7 (E) and T21 (F) downregulated genes calculated with Metascape tool. X axis shows significance reported as −log10 *p* value (*p*). Supplementary Figure S4. Uncropped Western blots. Supplementary Table S1. NanoString pathway annotation at distinct time points of osteogenic differentiation. Total number of genes in each NanoString Pathway and number of modulated genes, as well as relative log2 fold changes, are reported. Supplementary Table S2. NanoString pathway annotation at distinct time points of RG108 treatment. Total number of genes in each NanoString Pathway and number of modulated genes, as well as relative log2 fold changes, are reported.
