# Supplementary material for: Deciphering the Transcriptional Metabolic Profile of Adipose-Derived Stem Cells During Osteogenic Differentiation and Epigenetic Drug Treatment
Source: Cells. 2025 Jan 17;14(2):135. doi: 10.3390/cells14020135 (PMC11763738; doi:10.3390/cells14020135)
Supplement: Supplementary file 1 [file cells-14-00135-s001.zip › Supplementary Figure Legends.pdf]

Supplementary Figure S1. Nanostring pathways annotation of modulated genes during ASC osteogenic differentiation. Bar plots showing modulated genes within Nanostring annotated pathways at T7 (A), T14 (B) and T21 (C) compared to T0. Every square represents a single gene. Pathways were reported when at least 3 genes were modulated. Color bars show downmodulated (blue) and upregulated (red) genes.

Supplementary Figure S2. Nanostring pathways annotation of modulated genes during RG108 treatment compared to standard ASC osteogenic differentiation. Bar plots showing modulated genes within Nanostring annotated pathways at T7 (A) and T21 (B) compared to T0. Every square represents a single gene. Pathways were reported when at least 3 genes were modulated. Color bars show downmodulated (blue) and upregulated (red) genes, reported as $\log_2$ fold change.

Supplementary Figure S3. Transcriptional changes during osteogenesis differentiation of ASCs treated with RG108. River plots of upregulated genes at T7 (A) and at T21 (B), and downregulated genes at T7 (C) and T21 (D) of RG108 treatment compared to DMSO-treated cells. Graph bars of enriched pathways for RG108 T7 (E) and T21 (F) downregulated genes calculated with Metascape tool. X axis shows significance reported as -log10 p value (P).

Supplementary Figure S4. Uncropped Western blots.
