# Supplementary figures and images for: Deciphering the Transcriptional Metabolic Profile of Adipose-Derived Stem Cells During Osteogenic Differentiation and Epigenetic Drug Treatment
Source: Cells. 2025 Jan 17;14(2):135. doi: 10.3390/cells14020135 (PMC11763738; doi:10.3390/cells14020135)

**A**

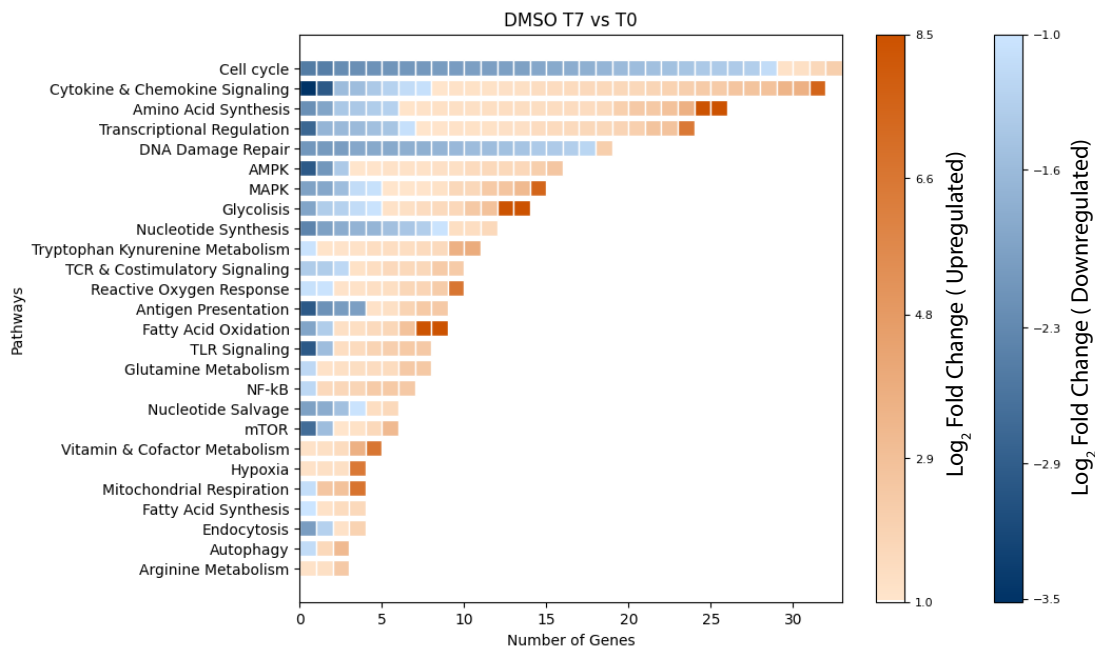

**B**

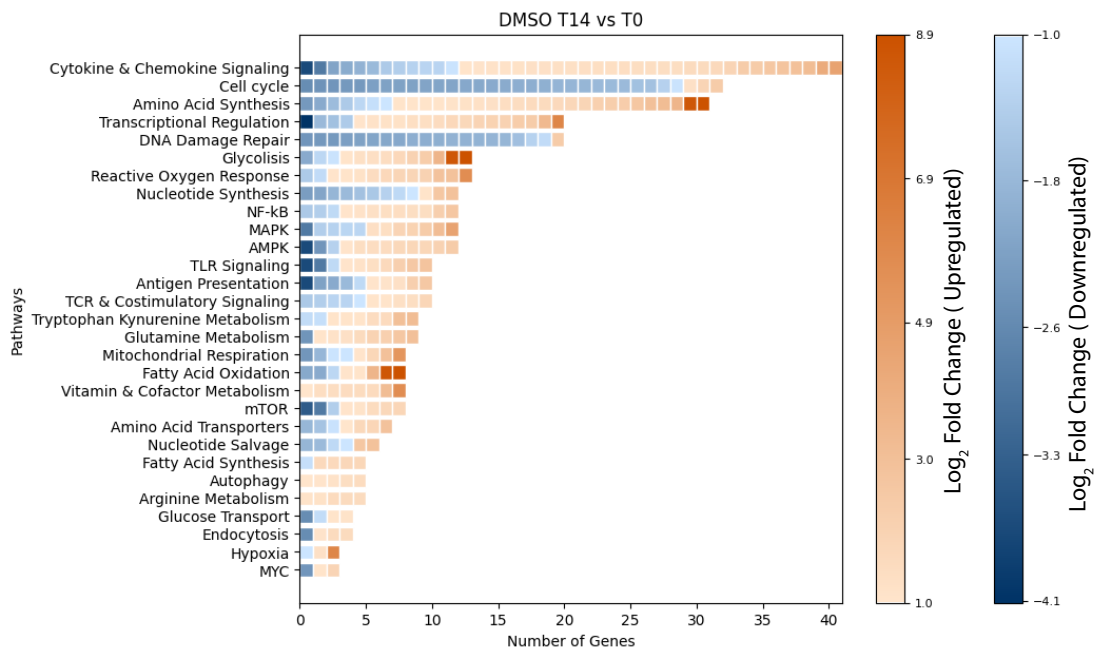

**C**

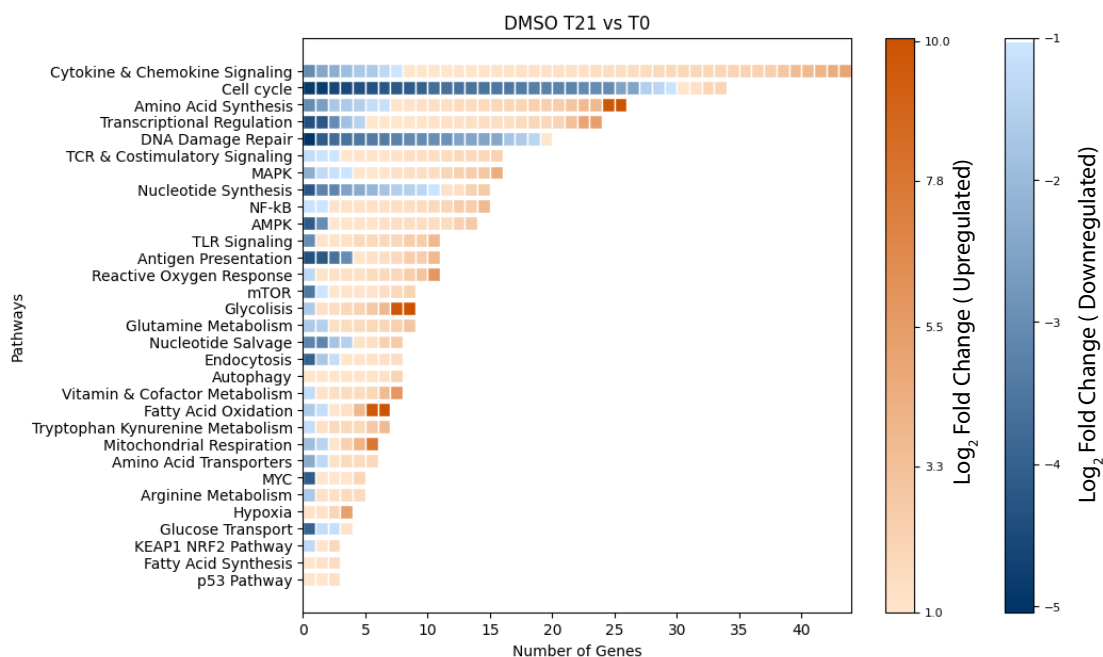

Supplement: Supplementary file 1 [file cells-14-00135-s001.zip › Supplementary Figure S1.pdf]

**A**

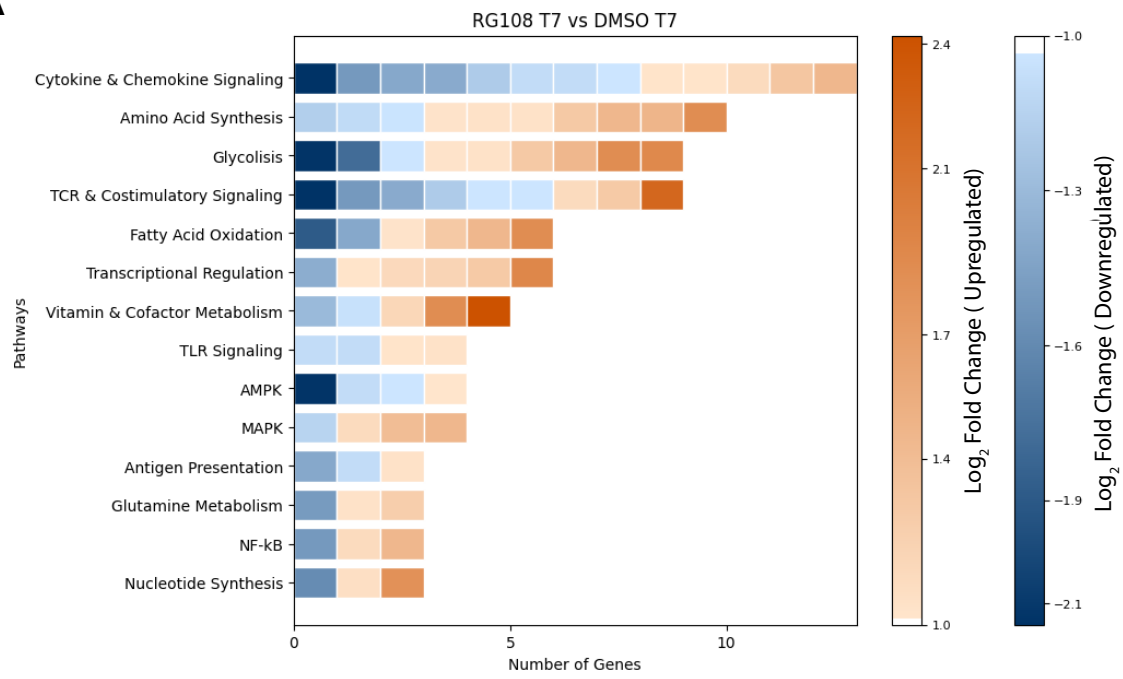

**B**

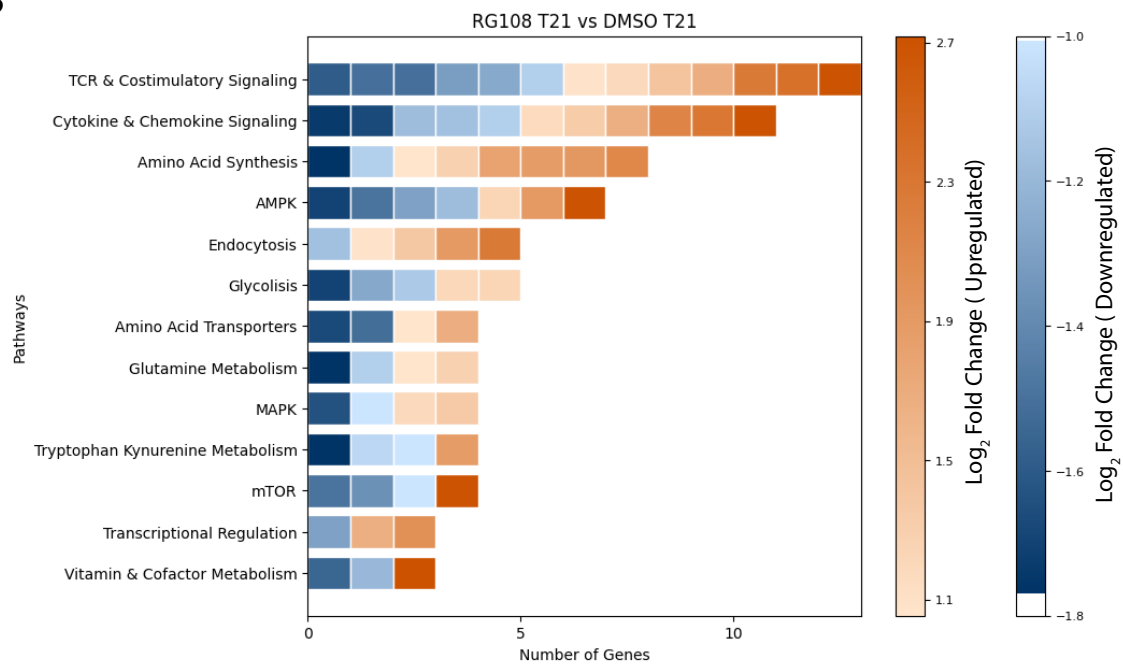

Supplement: Supplementary file 1 [file cells-14-00135-s001.zip › Supplementary Figure S2.pdf]

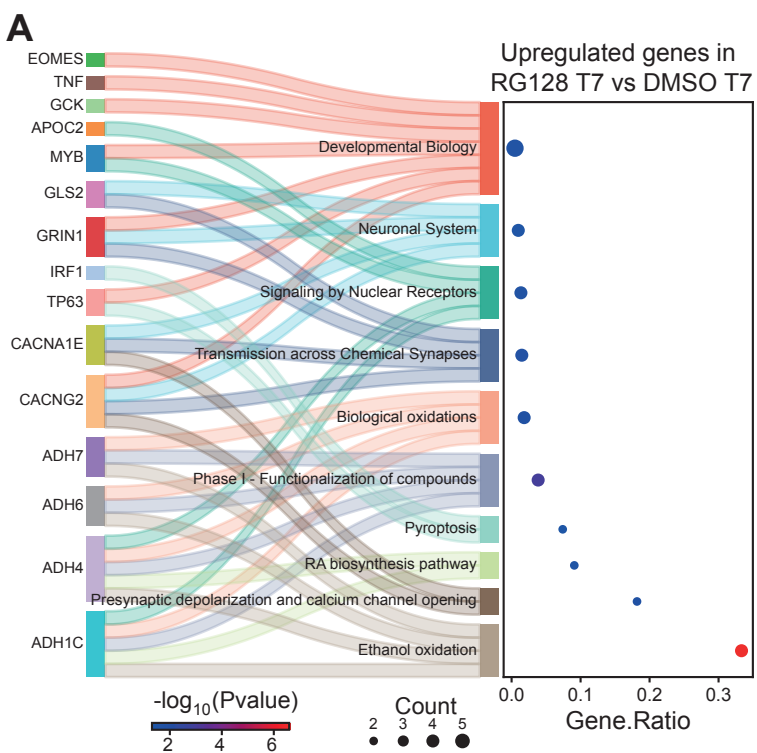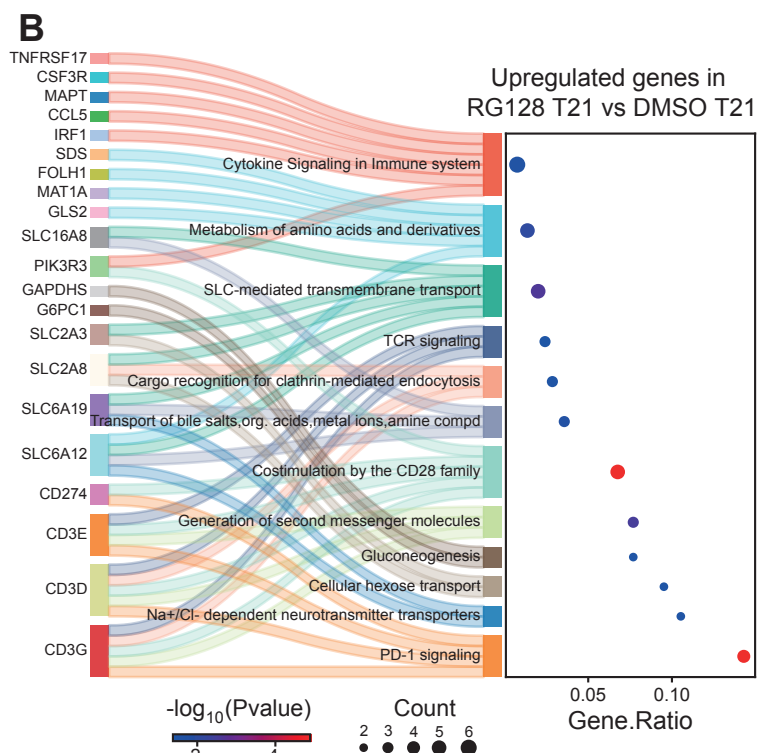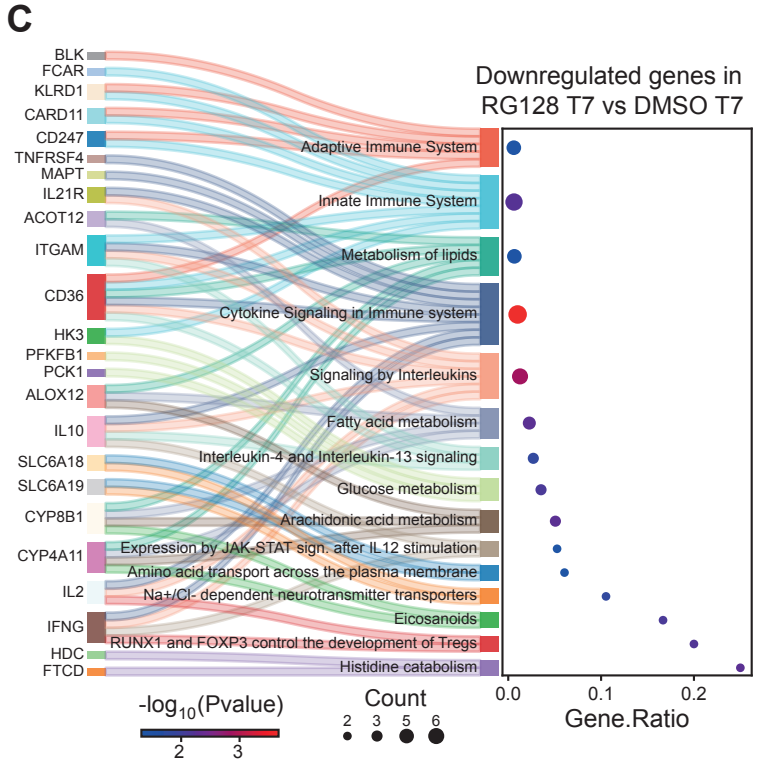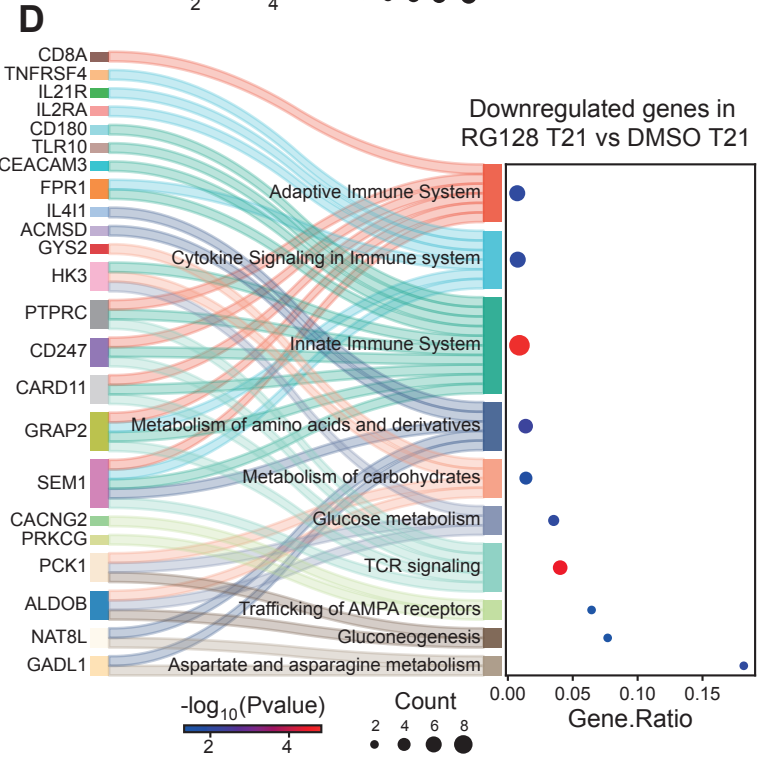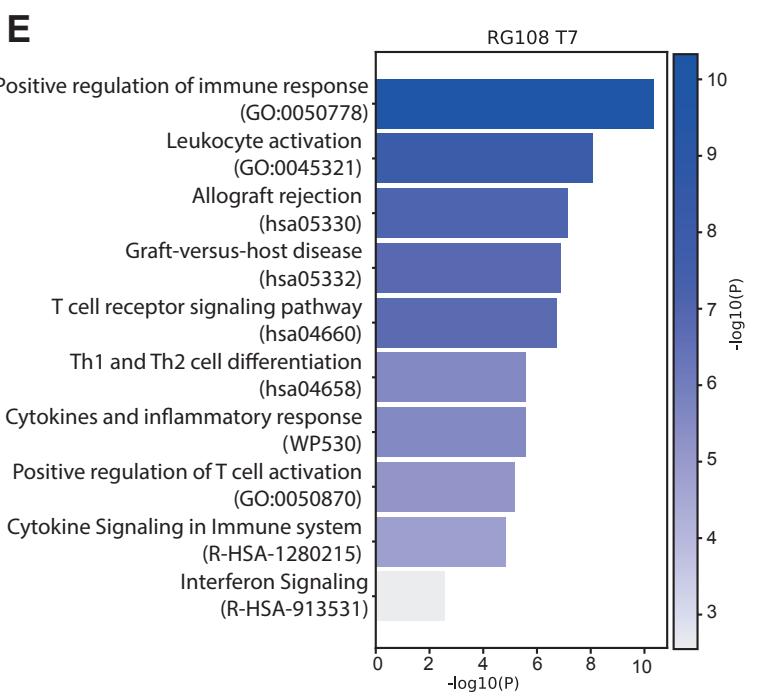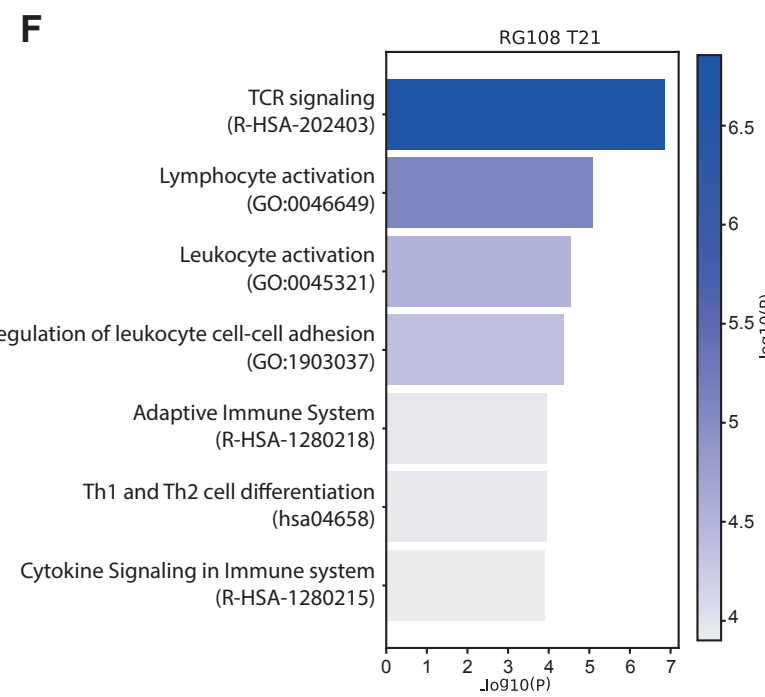

Supplement: Supplementary file 1 [file cells-14-00135-s001.zip › Supplementary Figure S3.pdf]

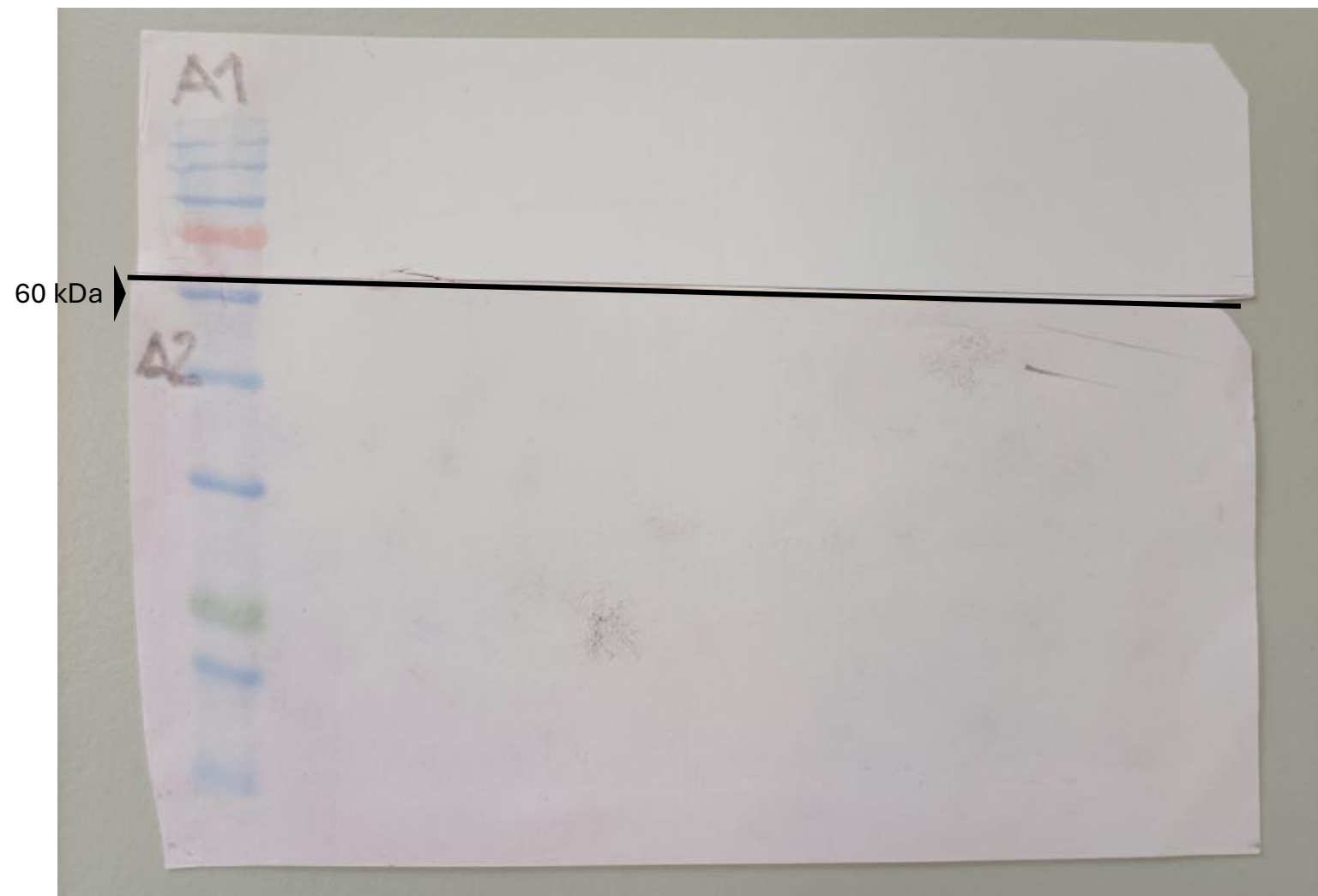

Supplement: Supplementary file 1 [file cells-14-00135-s001.zip › Supplementary Figure S4.pdf]
